# Atypical B cells and impaired SARS-CoV-2 neutralization following heterologous vaccination in the elderly

**DOI:** 10.1016/j.celrep.2023.112991

**Published:** 2023-08-16

**Authors:** Isabella A.T.M. Ferreira, Colin Y.C. Lee, William S. Foster, Adam Abdullahi, Lisa M. Dratva, Zewen Kelvin Tuong, Benjamin J. Stewart, John R. Ferdinand, Stephane M. Guillaume, Martin O.P. Potts, Marianne Perera, Benjamin A. Krishna, Ana Peñ alver, Mia Cabantous, Steven A. Kemp, Lourdes Ceron-Gutierrez, Soraya Ebrahimi, The CITIID-NIHR BioResource COVID-19 Collaboration, Paul Lyons, Kenneth G.C. Smith, John Bradley, Dami A. Collier, Laura E. McCoy, Agatha van der Klaauw, James E.D. Thaventhiran, I. Sadaf Farooqi, Sarah A. Teichmann, Paul A. MacAry, Rainer Doffinger, Mark R. Wills, Michelle A. Linterman, Menna R. Clatworthy, Ravindra K. Gupta

**Affiliations:** 1 Cambridge Institute of Therapeutic Immunology and Infectious Disease (CITIID), Cambridge, UK; 2 Department of Medicine, University of Cambridge, Cambridge, UK; 3 Molecular Immunity Unit, Department of Medicine, Medical Research Council Laboratory of Molecular Biology, University of Cambridge, Cambridge, UK; 4 Cellular Genetics, Wellcome Sanger Institute, Cambridge, UK; 5 Immunology Programme, Babraham Institute, Babraham Research Campus, Cambridge, UK; 6 Department of Clinical Biochemistry and Immunology, Cambridge University Hospital NHS Trust, Cambridge, UK; 7 Division of Infection and Immunity, UCL, London, UK; 8 University of Cambridge Metabolic Research Laboratories and NIHR Cambridge Biomedical Research Centre, Wellcome-Medical Research Council (MRC) Institute of Metabolic Science, Cambridge, UK; 9 MRC Toxicology Unit, University of Cambridge, Cambridge, UK; 10 National University of Singapore, Singapore, Singapore

## Abstract

Suboptimal responses to a primary vaccination course have been reported in the elderly, but there is little information regarding the impact of age on responses to booster third doses. Here, we show that individuals 70 years or older (median age 73, range 70–75) who received a primary two-dose schedule with AZD1222 and booster third dose with mRNA vaccine achieve significantly lower neutralizing antibody responses against SARS-CoV-2 spike pseudotyped virus compared with those younger than 70 (median age 66, range 54–69) at 1 month post booster. Impaired neutralization potency and breadth post third dose in the elderly is associated with circulating “atypical” spike-specific B cells expressing CD11c and FCRL5. However, when considering individuals who received three doses of mRNA vaccine, we did not observe differences in neutralization or enrichment in atypical B cells. This work highlights the finding that AdV and mRNA COVID-19 vaccine formats differentially instruct the memory B cell response.

## Graphical abstract

**Graphical abstract:**
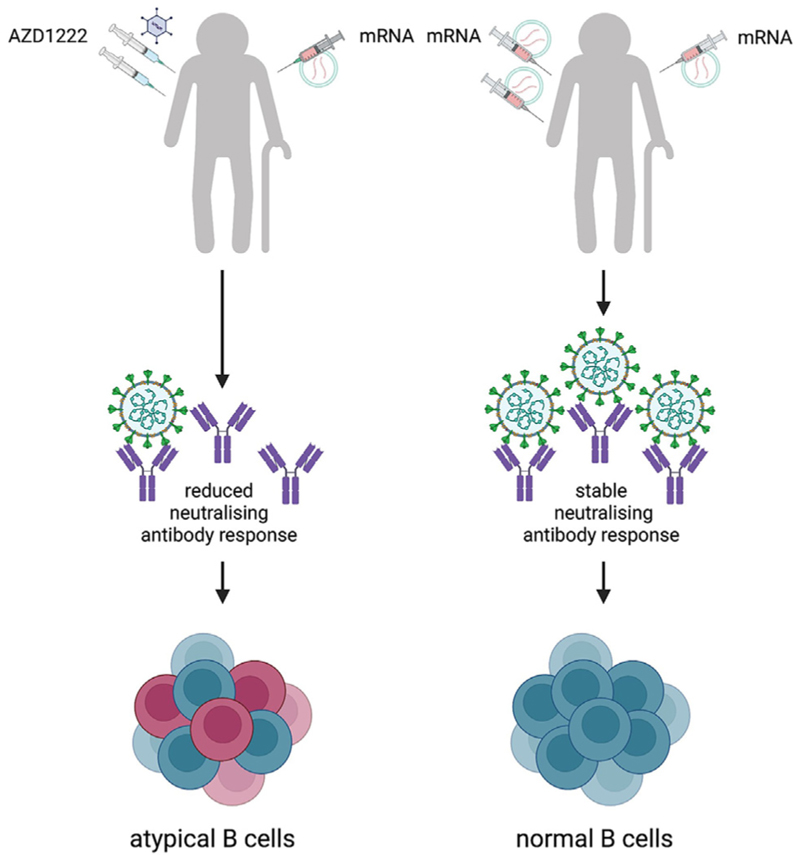

## Introduction

The adenovirus vectored AZD1222 vaccine (ChAdOx1 nCoV-19) was one of the first vaccines approved for use in the United Kingdom in early 2021,^1^ and came shortly after rollout of the Pfizer-BioNTech mRNA vaccine BNT162b2.^2^ During initial scale-up of vaccination in early 2021, there were several variants of concern circulating, including Alpha (B.1.1.7) and Beta (B.1.351)^3^; vaccines were shown to confer protection to Alpha but not Beta,^4–6^ likely due to escape from neutralizing antibodies mediated by the spike mutation E484K.^7^

With emergence of the Delta variant^8–10^ coupled with waning neutralizing antibodies,^11,12^ booster doses were recommended.^13^ Emergence of the Omicron BA.1 variant^14^ further strengthened the argument for booster doses when data emerged showing broader neutralization compared with two doses.^15–17^ In contrast to neutralizing antibody titers, spike-specific B cell frequencies remain stable across time, and after the third dose neutralizing antibodies appear more able to tolerate receptor-binding domain (RBD) mutations, consistent with ongoing antibody maturation.^18–20^

Long-lived B cell immunity, important in maintaining immunity elicited by vaccines,^20,21^ is affected by immune aging in the elderly and, moreover, functional recall to SARS-CoV-2 is lower than in younger individuals.^22–26^ Our previous work indicated that age broadly affected immune responses in those vaccinated with the mRNA vaccine BNT162b2,^27^ particularly following first SARS-CoV-2 vaccine dose. This difference diminished after the second dose of the vaccine, but the T cell response remained poorer in the elderly despite the second mRNA vaccine dose.

Here, we aimed to determine the impact of age on responses to the third vaccine dose and to understand the mechanistic underpinning of the differential immune responses observed with increasing age.^27^ In the UK, individuals vaccinated with AZD1222 received either the BNT162b2 or mRNA-1273 vaccine boosting approximately 6 months after their second dose.^28^ We focused on individuals who received two doses of AZD1222 and an mRNA booster vaccine because we and others have reported lower neutralizing antibody responses following two doses of AZD1222 compared with BNT162b2.^8,15,29,30^ We measured the breadth and durability of vaccine-elicited neutralizing antibody and T cell responses across 36 individuals receiving AZD1222 as their primary two-dose course. We also applied multiparameter flow cytometry and single-cell RNA sequencing (scRNA-seq) to peripheral blood mononuclear cells (PBMCs) obtained 1 month following the second dose of AZD1222, and 1 month after the BNT162b2 booster dose, comparing cell phenotypes, single-cell transcriptomes, and antigen receptor sequences longitudinally across age groups. We compared serum-neutralizing activity with individuals who received the BNT162b2 primary two-dose course plus mRNA vaccine as the third dose.

## Results

### Binding and neutralizing antibody responses following two doses of AZD1222 and third dose of mRNA vaccination

**Figure 1:**
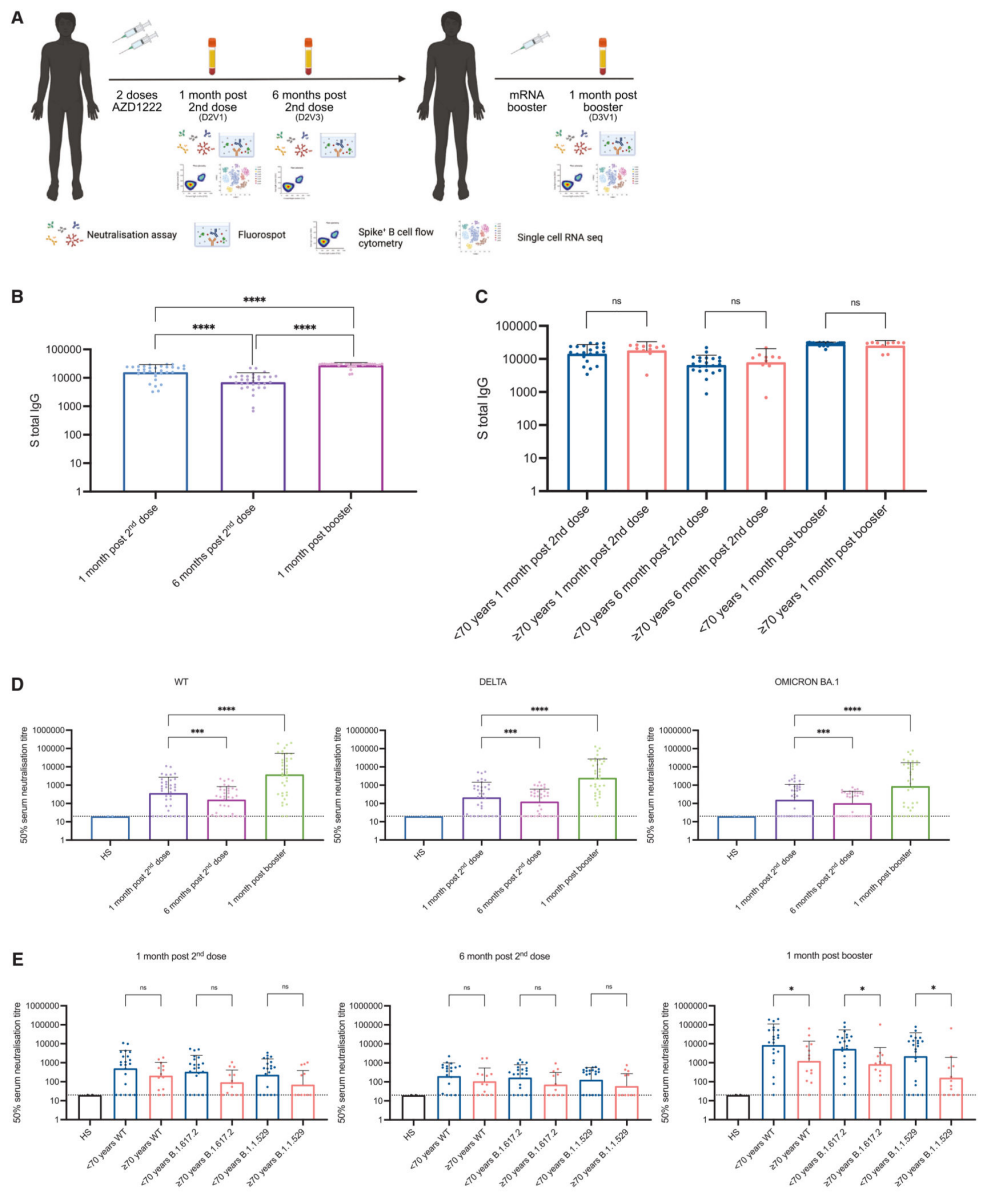
Longitudinal neutralizing plasma antibody titers against SARS-CoV-2 Wu-1 D614G WT, Delta, and Omicron BA.1 variants from AZD1222 vaccinated individuals boosted with an mRNA-based vaccine (A) Study design. Thirty-six individuals vaccinated with AZD1222 and boosted with an mRNA-based vaccine were recruited. Longitudinal blood draws occurred 1 month post second dose, 6 months post second dose, and 1 month post booster. (B) Total anti-spike IgG binding antibody responses at 1 month post second dose, 6 months post second dose, and 1 month post booster. Wilcoxon matched-pairs signed-rank test was used. ****p < 0.0001. (C) Total anti-spike IgG binding antibody responses at 1 month post second dose, 6 months post second dose, and 1 month post booster stratified by those below age 70 years and those age 70 and above. Mann-Whitney test was used. ns, not significant. (D) Neutralization titers (ID_50_) of sera were measured against Wu-1D614GWT, Delta, and Omicron for each time point. A Wilcoxon matched-pairs signed-rank test was used to determine significance in titers between time points. **p < 0.01, ***p < 0.001, ****p < 0.0001. (E) Neutralization titers (ID_50_) against Wu-1 D614G WT, Delta, and Omicron BA.1 stratified by those below age 70 years and those age 70 and above. Mann-Whitney test was used. ns, not significant; *p < 0.05, **p < 0.01. HS denotes human serum from unvaccinated, unexposed individuals collected prior to the SARS-CoV-2 pandemic. Data are representative of two individual experiments across 36 donor samples. Each experiment contained a technical repeat.

We enrolled 60 individuals who had been vaccinated with two doses of AZD1222 and one mRNA booster vaccine (either BNT162b2 or mRNA-1273). Blood draws were taken 1 month post second dose, 6 months post second dose, and 1 month post booster third dose (Figures 1A and S1A). Thirty-six individuals had samples available for all time points and were N-antibody-negative at all time points, indicating no natural infection in these individuals (Table S1 and Figure 1B). The median age of study participants was 67 years. Individuals were stratified into those <70 years of age (median = 66 [62–68]) and those ≥70 (median = 73 [70–74]) (Table S1). There was no statistically significant difference between these age groups with regard to prevalence of diabetes or history of immune suppression/cancer or kidney disease. Cardiovascular disease was more common in those ≥70, as expected. We initially measured SARS-CoV-2 spike (S) total immunoglobulin G (IgG) along with N total IgG using Luminex-based flow-cytometric analysis,^31^ the latter to exclude any individuals who may have had SARS-CoV-2 infection from our study (Figure S1A). It should be noted, however, that N antibody titers wane, in some cases over short periods.^32,10^

Total S IgG, as measured by mean fluorescence intensity (MFI), decreased between 1 month and 6 months post second dose of AZD1222 (p < 0.0001), with a significant increase evident following the booster mRNA vaccination (p < 0.0001) (Figure 1B). A significant increase was also present when comparing 1 month post second dose and 1 month post booster (p < 0.0001) (Figures 1B and S1B), indicating that the booster had an additive effect on S total IgG. When comparing <70- and ≥70-year-old age groups, there was no significant difference in S total IgG at any time point (Figures 1C and S2A–S2C).

We assessed neutralizing antibodies using a previously developed spike-pseudotyped lentiviral neutralization assay.^27^ SARS-CoV-2 D614G wild-type (WT) spike was used as the comparator spike against the Delta and Omicron BA.1 variants of concern. Overall, geometric mean titers (GMT) as a measure of the mean ID_50_ at each time point showed a decrease for WT from 1 to 6 months post second dose (GMT = 371.4, standard deviation [SD] 7.33 and 163.1 [SD 5.14], respectively), but a robust augmentation 1 month following the booster mRNA vaccine dose (GMT = 3,849, SD 14.23), (Figures 1D and S3A).

Across the ancestral D614G, Delta, and Omicron variants, there was a significant decrease in neutralizing antibodies 6 months post second dose compared with 1 month post second dose (p < 0.0002, p < 0.0005, and p < 0.0001 for D614G, Delta, and Omicron, respectively). Fold changes indicated relatively modest waning in circulating neutralizing antibodies against WT and Delta between 1 month post second dose and 6 months post second dose (Figure 1D). A greater degree of waning was observed for Omicron (Figure 1D). Boosting with an mRNA-based vaccine showed a significant increase in neutralizing antibodies across the three variants compared with 1 month post second dose (103-fold increase between post second dose and post booster for WT, 29-fold increase for Delta, and 19-fold increase for Omicron) (Figure 1D). Human serum obtained prior to the pandemic from unexposed, unvaccinated individuals was used as a negative control.^33^

We next assessed the impact of age on boosting of neutralizing antibody responses. No differences in serum-neutralizing antibody titers were observed across age groups for the time points of 1 month post second dose and 6 months post second dose across WT and the two variants of concern (Figures 1E and S3B–S3F). As expected, there was a log decrease in neutralizing antibody titers between 1 month post booster and 6 months post booster. However, the ≥70-year-old group (median age 73, range 70–75) demonstrated significantly lower neutralizing antibody GMTs 1 month post booster compared with those <70 years old (median age 67, range 52–69) (Delta: p < 0.011; Omicron: p < 0.021). After the mRNA booster vaccine, 4% of individuals <70 years old were non-neutralizers (ID_50_ titers of <20) and 8% of ≥70-year-olds were non-responders for WT. For Delta, 4% of <70-year-olds were non-neutralizers and 15% of ≥70-year-olds compared with 17% of <70-year-olds and 22% of ≥70-year-olds for Omicron (Figure 1E). In summary, the mRNA booster elicits a robust augmentation in neutralizing antibodies, with a diminished response in participants aged 70 years or older.

### Virus-specific atypical B memory cells expanded in the elderly post mRNA booster

**Figure 2:**
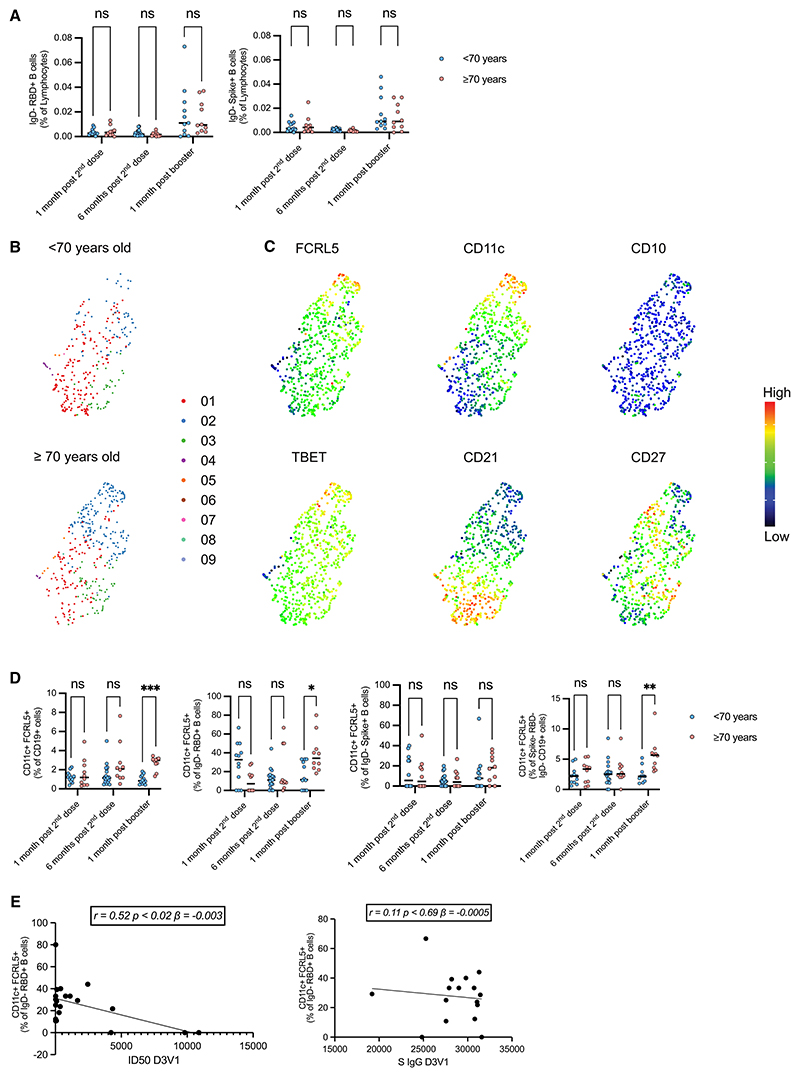
Older individuals have a higher frequency of antigen-specific atypical B cells after mRNA vaccine booster (A) IgD^−^RBD^+^ and IgD^−^Spike^+^ B cell frequency, (as a percentage of live, single lymphocytes) at each time point; multiple Mann-Whitney tests per row with Holm-šidák multiple testing correction was used. (B) Uniform manifold approximation projection (UMAP) clustering analysis of a subset of IgD^−^RBD^+^ B cells from D3V1. (C) Relative MFI of indicated markers in UMAP clustering analysis from (B). (D) Atypical (CD11c^+^FCRL5^+^) B cell frequency, (as a percentage of CD19^+^ cells, IgD^−^RBD^+^, and IgD^−^Spike^+^ cells, respectively) at each time point. D2V1, 1 month post second dose; D2V3, 6 months post second dose; D3V1, 1 month post booster. Each symbol represents a unique biological sample; multiple Mann-Whitney tests per row with Holm-šidák multiple testing correction was used. (E) Correlation between neutralization ID_50_ or binding-spike-specific IgG and percentage of atypical B cells. Data are representative of two individual experiments across 36 donor samples.

To investigate the antigen-binding capacity of memory B cells, we phenotypically assessed circulating SARS-CoV-2 RBD- and spike-binding B cells by high-content spectral cytometry.^30^ Overall, there was an increase in the proportional representation of both RBD- and spike-binding non-naive (IgD^−^) B cells among lymphocytes 1 month post mRNA vaccine booster compared with 1 and 6 months post second dose of AZD1222, which was comparable in subjects <70 and ≥70 years of age (Figure 2A). However, unbiased uniform manifold approximation and projection (UMAP) machine-learning analysis showed an altered distribution of IgD^−^ spike-binding B cell subsets between the <70- and ≥70-year-old groups (Figures 2B and S4). One such subpopulation expanded in the ≥70-year-old group had increased expression of FcRL5, CD11c, and TBET, with low expression of CD21 and CD27, consistent with an atypical memory B cell phenotype (Figure 2C). A distinct population of CD11c^+^FcRL5^+^ atypical B cells was evident using conventional biaxial gating (Figures S4D–S4F). When considering RBD- and spike-binding non-naive B cells at this time point, there was also a greater proportion of antigen-specific atypical non-naive B cells in older subjects compared with younger subjects, with an average of 39% of IgD^−^RBD^+^ B cells having atypical phenotype within the ≥70-year-old group (p < 0.038 for RBD, Figures 2D and S4), as compared with 10% in the <70-year-old group. Of note, we did not find a statistically significant linear correlation between age and atypical B cells (Figure S5).

To ascertain whether the presence of atypical cells was linked with poorer neutralization, we performed correlation analysis between atypical FcRL5, CD11c^+^ B cells as a proportion of IgD^−^RBD^+^ B cells and (1) binding anti-spike IgG titers and (2) neutralization titers. We observed no relationship between atypical B cell abundance and serum IgG but a significant negative correlation with serum-neutralizing activity (Figure 2E). Taken together, our data suggest that the mRNA vaccine booster is able to support the expansion of vaccine-specific memory B cells, but that being older than 70 years is associated with a skewed B cell differentiation toward atypical memory B cells that generate lower-potency neutralizing antibodies, consistent with previous reports regarding their lower effectiveness at contributing to protective humoral immunity.^34–36^

### Serum neutralization and atypical memory B cell response after three doses of BNT162b2

**Figure 3:**
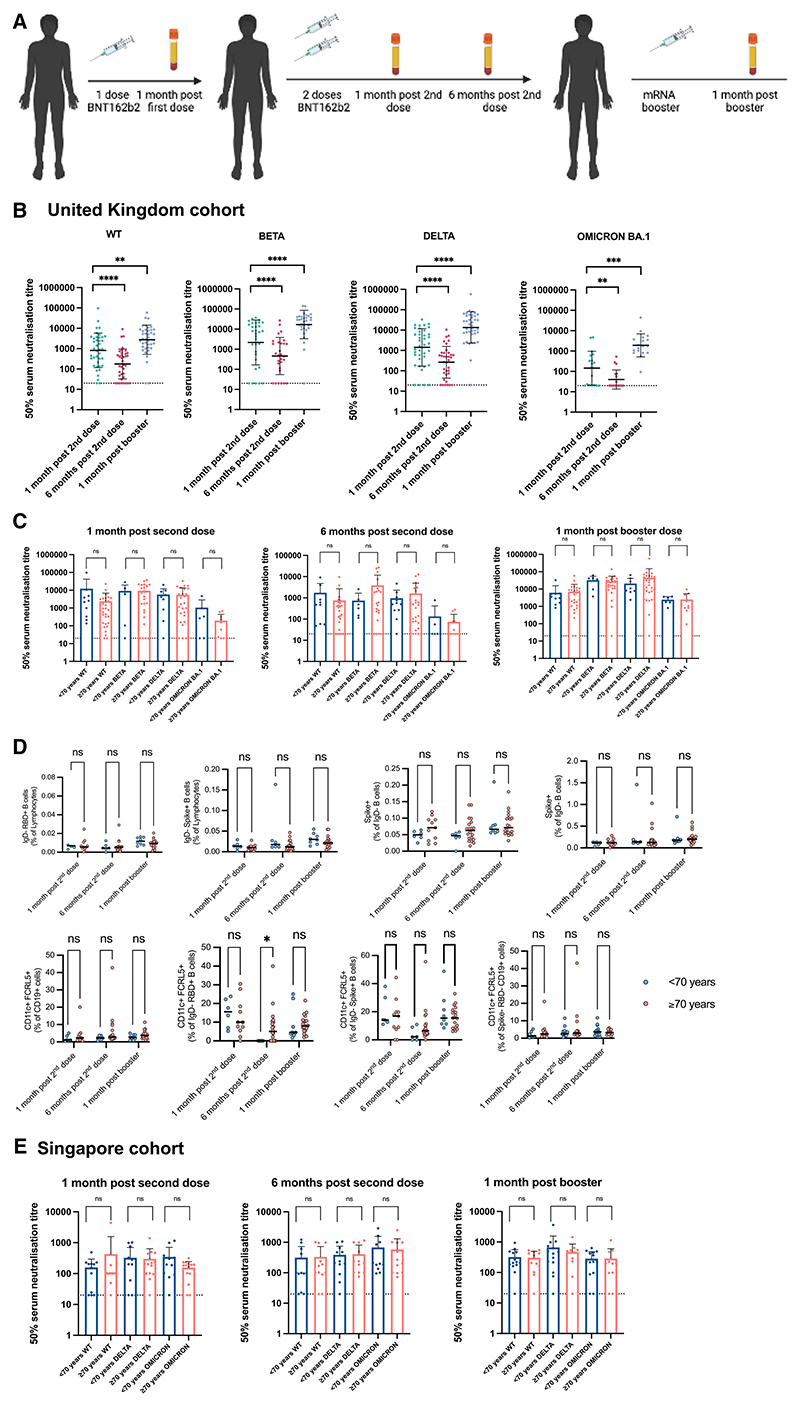
Longitudinal neutralizing plasma antibody titers against SARS-CoV-2 Wu-1 D614G WT, Beta, Delta, and Omicron BA.1 variants from BNT162b2 triple-vaccinated individuals (A) Study design. N-antibody-negative individuals vaccinated in the United Kingdom and 20 individuals vaccinated in Singapore with three doses of BNT162b2 were recruited. Longitudinal blood draws occurred at 1 month post second dose, 6 months post second dose, and 1 month post booster. (B) Neutralizing antibody data against WT, Beta, Delta, and Omicron BA.1. A Wilcoxon matched-pairs signed-rank test was used to determine significance between time points. **p < 0.01, ***p < 0.001, ****p < 0.0001. (C) Neutralizing antibody data stratified by age into those below age 70 and those age 70 and above. Mann-Whitney test was used. ns, not significant. (D) Proportions of B cell subsets 1 month post dose 2 (left) and 1 month post mRNA booster (right) in individual study participants in different age groups. Significance testing using Kruskal-Wallis one-way test. (E) Neutralizing antibody data stratified by age against WT, Delta, and Omicron in individuals vaccinated in Singapore with three doses of BNT162b2 (Table S2). Data are representative of two individual experiments across 38 donor samples.

We sought to understand whether the phenomenon of age-related increases in atypical B cells with impaired serum neutralization was related to primary two-dose vaccination with adenovirus vectored AZD1222 or also a feature of vaccination with mRNA BNT162b2 primary two-dose vaccination. Alongside the AZD1222 vaccinee cohort, we recruited individuals from the same underlying community who had been vaccinated with three doses of BNT162b2 (Figure 3A) and who were N antibody negative from pre-vaccine to dose-3 time points. These individuals were older than the AZD1222 recipients, with a median age of 73 years and consistent with BNT162b2 use prioritized in the over-80 age group before AZD1222 in the UK. Significant neutralizing antibody waning was observed after 6 months post second dose compared with 1 month post second dose across D614G WT (p < 0.0001), Delta (p < 0.0001), and Omicron BA.1 (p < 0.0081) (Figure 3B). When stratified by age, no significant differences between participants <70 and ≥70 years old were observed at any of the time points, nor for any of the variants (Figure 3C). Similarly, no age-related differences in neutralization following dose 3 were observed in a cohort of BNT162b2 vaccinated individuals recruited in Singapore (Figure 3E and Table S2). These data indicate that neutralizing antibodies elicited by three doses of the BNT162b2 vaccine are not affected by age. We additionally measured the frequency of atypical B cells (CD11c^+^FcRL5^+^) in individuals vaccinated in the UK with three doses of BNT162b2. In contrast to individuals primed with two doses of AZD1222 and an mRNA booster, no significant age effects were observed in the frequency of atypical B cells in individuals vaccinated with three doses of BNT162b2 (Figure 3D).

Of note, the median ages in the “old” versus “young” age groups differed by only 7 years (Figure S1). We therefore performed a sensitivity analysis by including a set of 15 younger “control” individuals below the age of 60 years vaccinated with either of the two regimens (2×AZD1222 + BNT162b2 or 3× BNT162b2). The participants were from the same underlying UK population and the same methodology for blood sampling, storage, and cellular phenotyping was used. The main results were not affected by inclusion of these younger individuals (Figure S6); the 2×AZD1222 + BNT162b2 vaccinated individuals (median age of <70-year-old group with additional younger participants was 65), and not those vaccinated with 3×BNT162b2, showed an age-related defect in neutralization that was associated with frequency of atypical virus-specific B cells.

### scRNA-seq identifies age-related differences in B cell vaccine response

**Figure 4:**
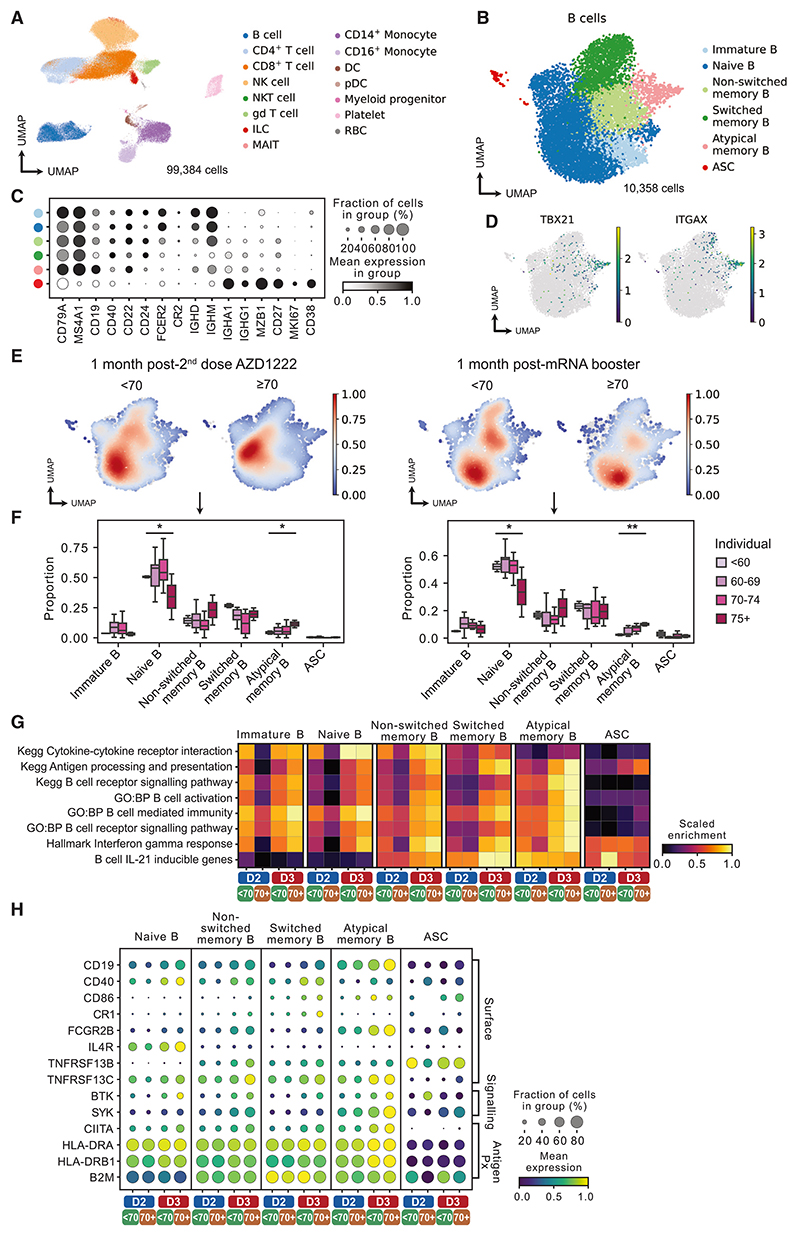
Single-cell RNA-seq identifies age-associated differences in B cell responses post vaccination (A) UMAP of cell types identified by scRNA-seq of PBMCs in samples taken 1 month post dose 2 AZD1222 (n = 20 subjects) and 1 month post mRNA booster (n = 19 subjects). (B and C) UMAP (B) of subsetted B cells annotated by canonical marker gene expression (C). (D) Atypical memory B cells express *TBX21* and *ITGAX*. (E) Density plots showing B cell abundance in <70-year-old and ≥70-year-old individuals 1 month post dose 2 AZD1222 (left) and 1 month post mRNA booster (right). (F) Proportions of B cell subsets 1 month post dose 2 AZD1222 (left) and 1 month post mRNA booster (right) in individual study participants in different age groups. Significance testing using Kruskal-Wallis one-way test. (G) Heatmap showing gene set expression in B cell subsets in <70-year-old and ≥70-year-old individuals 1 month post dose 2 AZD1222 (D2) and 1 month post mRNA booster (D3). (H) Selected differentially expressed genes driving differences in (G). Table S1 shows a list of individuals included in the scRNA-seq analysis.

To further investigate the nature of reduced immune responses in the elderly following heterologous vaccination, we performed scRNA-seq to assess gene expression, as well as single-cell B cell receptor (BCR) and T cell receptor (TCR) sequencing, in PBMCs taken 1 month post dose 2 of AZD1222 (n = 20 participants) and 1 month post mRNA booster (n = 19 participants). Following the application of a rigorous quality control pipeline, 99,384 cells were available for analysis, and annotated using CellTypist^37^ and canonical marker gene expression, identifying 15 major cell types including CD4 and CD8 T cells, B cells, monocytes (classical and non-classical), classical dendritic cells (DCs), plasmacytoid DCs (pDCs), natural killer (NK) cells, innate lymphoid cells (ILCs), and mucosal-associated invariant T cells (MAITs) (Figures 4A and S7).

When considering the B cell compartment in isolation, fine clustering identified a small number of antibody-secreting cells as well as immature, naive, non-switched, and switched memory B cells and a population of *TBX1*-expressing (encoding TBET) and *ITGAX*-expressing (encoding CD11c) “atypical” memory B cells (also previously described as exhausted or age-associated B cells^34^) (Figures 4B–4D and S7A–S7C). The abundance of naive B cells was lowest in those ≥70 years of age, and there was an increase in atypical memory B cells with increasing age, both following dose 2 of AZD1222 and mRNA booster vaccines (Figures 4E and 4F).

Pathway enrichment analysis showed differences between vaccine doses and according to age. Overall, the magnitude of expression of several relevant gene sets across B cell subsets was greater at 1 month following the mRNA booster (D3) compared with the same time point post dose 2 of AZD1222 (D2) (Figure 4G). This difference was particularly marked in antigen-experienced subsets (memory and atypical B cells); for example, “antigen processing and presentation” pathway genes, such as *CD40*, were minimally expressed on these cell subsets post D2 but demonstrated robust expression post D3 (Figures 4G, 4H, and S8A–S8C). “Cytokine-cytokine receptor interaction” genes were also increased post D3 compared with D2, particularly in naive and non-switched memory B cells, and included *IL4R* and BAFF receptors *TNFRSF13B* (encoding TACI) and *TNFRSF13C* (encoding BAFF-R) (Figures 4G, 4H, S8D, and S8E). “Interferon gamma response” and “IL-21 inducible genes” were increased following D3, which are important for class switch recombination and B cell persistence in the germinal center, respectively.^38^

Notably, the difference in gene set enrichment between D2 and D3 samples was more marked in the ≥70-year-old age group. For example, in naive B cells post D2, “cytokine-cytokine receptor interaction” genes showed modest expression in those aged <70 but were barely detectable in cells from participants aged ≥70. In contrast, post D3 this gene set was robustly expressed at similar levels in both age groups (Figure 4G). Indeed, in atypical B cells post D3, enrichment of B cell activation pathways in the ≥70-year-old age group was significantly higher than that of younger subjects (Figures 4G, S8D, and S8E), suggesting that the atypical B cell population is not only expanded but also more activated in the elderly age group post D3. Of note, interferon-γ (IFN-γ) has been shown to drive the expansion of atypical memory B cells in the context of malaria infection,^39^ and here we found a greater enrichment of “interferon gamma response” genes across all memory B cell subsets post D3 in the ≥70-year-old age group compared with those <70 years old, suggesting that this may underpin the age-associated expansion in atypical B cells in this context.

### T cell responses following two doses of AZD1222 and third-dose mRNA vaccination

**Figure 5:**
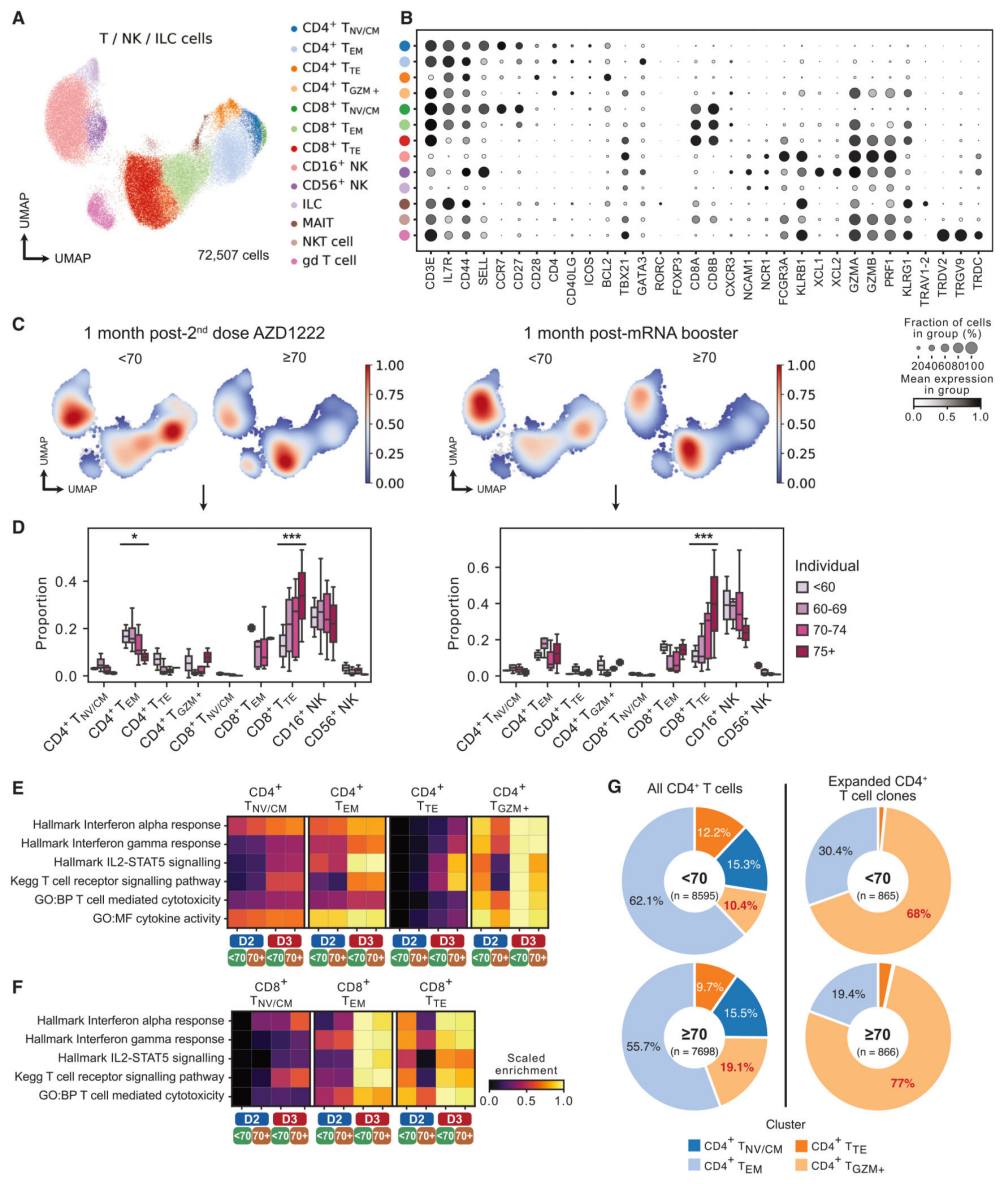
T cell responses to two doses of AZD1222 and an mRNA booster (A and B) UMAP of subsetted T cells, natural killer cells (NK), and innate lymphoid cells (ILC) (A) annotated by canonical marker gene expression (B). (C) Density plots showing T/NK/ILC cell abundance in <70-year-old and ≥70-year-old individuals 1 month post dose 2 AZD1222 (left) and 1 month post mRNA booster (right). (D) Proportions of T/NK/ILC cell subsets 1 month post dose 2 AZD1222 (left) and 1 month post mRNA booster (right) in individual individuals in different age groups. Significance testing using Kruskal-Wallis one-way test. (E and F) Heatmap showing gene set expression in CD4^+^ T cell subsets (E) and CD8^+^ T cell subsets (F) in <70-year-old and ≥70-year-old individuals 1 month post dose 2 AZD1222 (D2) and 1 month post mRNA booster (D3). (G) Proportions of CD4^+^ T cells and expanded CD4^+^ T cell clones in <70-year-old and ≥70-year-old individuals. Data are representative of an experiment with two technical repeats across 36 donor samples.

T cells are thought to maintain protection against SARS-CoV-2 infection when neutralizing antibody levels wane over time.^40^ We therefore considered the T cell and innate lymphocyte scRNA-seq transcriptomes in isolation, comprising 72,507 cells, including naive, effector memory (EM), terminal effector (TE), and cytotoxic CD4 T cells and naive, EM, and TE CD8 T cells, as well as CD16^+^ and CD56^+^CD16^−^ NK cells, ILCs, MAITs, NK T cells, and γδT cells (Figures 5A and 5B). There was a marked increase in abundance of TE CD8 T cells with increasing age, following both dose 2 of AZD1222 and mRNA booster vaccine (Figures 5C and 5D).

Pathway enrichment analysis showed marked differences in expression across CD4 T cell subsets between samples taken at 1 month following dose 2 of AZD1222 (D2) compared with the same time point post booster mRNA vaccine (D3) (Figure 5E). Expression of several relevant gene sets, for example, “interferon alpha response,” “interferon gamma response,” and “IL-2-STAT5 signaling” genes was greater post mRNA vaccine in all CD4 T cell subsets (Figures 5E and S9A). Indeed, among TE CD4 T cells, expression of these genes were low following dose 2 of AZD1222 in either age group; however, post mRNA vaccine there was a marked induction of “IL-2-STAT5 signaling” and “T cell receptor signaling” genes, particularly in the ≥70-year-old age group, including *CD44* and *CD69*, consistent with our previous work on vaccine-specific TE CD4 responses in older people.^41^ In the cytotoxic CD4 T cells, the ≥70-year-old age group showed muted expression of “interferon alpha response” and “interferon gamma response” gene sets post dose 2 of AZD1222 compared with the <70-year-old group. However, following the mRNA booster, both age groups showed a similar enrichment of these genes (Figures 5E and S9A).

In CD8 T cell subsets, several pathways were enriched 1 month post mRNA booster compared with 1 month following dose 2 of AZD1222 (D2) (Figure 5F). In addition, in TE CD8 T cells in particular, the more muted expression observed in the ≥70-year-old group post D2 was reversed by the mRNA vaccine, with similar expression observed in the ≥70-year-old group relative to the <70-year-old group, including *GZMA* (Figures 5F and S9B).

Analysis of single-cell TCR sequencing (scTCR-seq) data from CD4 T cells revealed several expanded TCR clones, which were enriched among *GZMA/B*-expressing cytotoxic CD4 T cells (Figures 4G and S9C). In elderly subjects, these cytotoxic CD4 T cells constituted a greater proportion of CD4 T cells as well as expanded CD4 T cell clones than in younger individuals. Of note, expansion of a cytotoxic CD4^+^ T cell subset has been associated with increased disease severity following SARS-CoV-2 infection but may also contribute to viral clearance.^42^

### Muted virus-specific T cell expansion and cytokine responses in the elderly post AZ prime and mRNA boost

**Figure 6:**
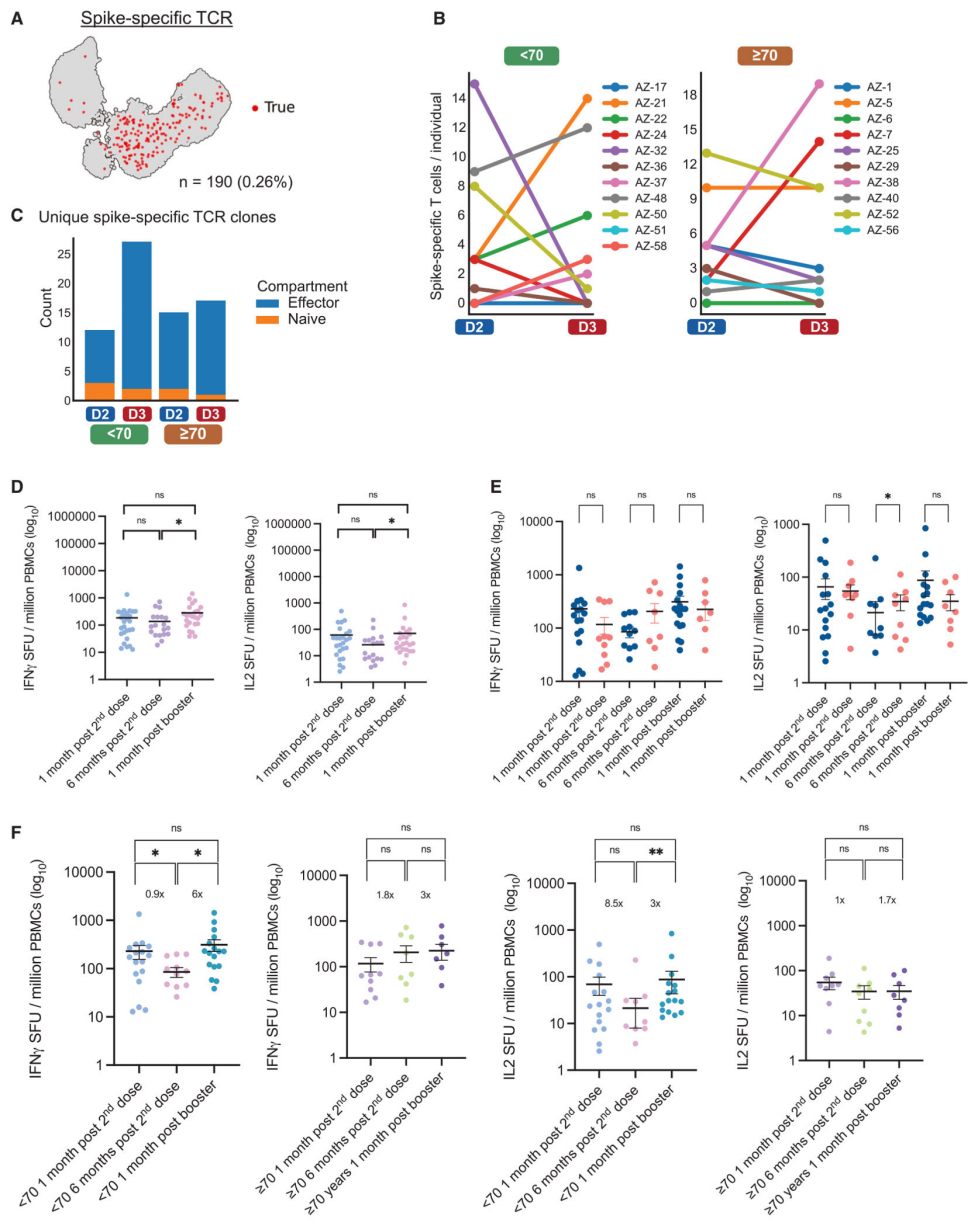
Age-associated changes in SARS-CoV-2 spike-specific circulating T cells following AZD1222 and an mRNA booster (A) Paired single-cell TCR (scTCR) CDR3 sequences with predicted specificity for epitopes derived from SARS-CoV-2 spike surface antigen. UMAP highlights n = 190 T cells with predicted binding capacity. (B) Frequency of spike epitope-specific scTCRs per individual 1 month post D2 and 1 month post mRNA booster (D3), separated by age group. (C) Sum of unique spike epitope-specific scTCR clones by corresponding T cell identity (effector or naive) across all individuals, at 1 month post-D2 and 1 month post mRNA booster (D3) in <70-year-old and ≥70-year-old age groups. (D) Fluorospot analysis of IFN-γ and IL-2 T cell responses to SARS-CoV-2 Wu-1 D614G WT at each longitudinal time point. Wilcoxon matched-pairs signed-rank test was used. ns, not significant; *p < 0.05. (E) IFN-γ and IL-2 SFUs per million PBMCs across longitudinal time points stratified by those below age 70 years and those age 70 and older. SFU, spot-forming units measured by Fluorospot assay. Significance testing using Mann-Whitney test (D and E). ns, not significant; *p < 0.05. (F) IFN-γ and IL-2 SFUs per million PBMCs by age group. Wilcoxon matched-pairs signed-rank test was used. ns, not significant; *p < 0.05, **p < 0.01. Data are representative of an experiment with two technical repeats across 36 donor samples.

To explore differences in SARS-CoV-2 spike antigen-specific circulating T cell responses following the different vaccination doses and between age groups, unsupervised clustering analysis was first applied to TCR repertoires across all subjects in whom scRNA-seq was performed (Table S3). We investigated the specificity of T cells to SARS-CoV-2 antigen-derived epitopes by comparing our scTCR-seq data with previously validated SARS-CoV-2-specific sequences from the Immune Epitope Database (IEDB) and VDJdb database.^43–45^ This led to the identification of 190 single-cell TCRs with putative spike epitope-binding capacity (Figure 6A). As expected, predicted SARS-CoV-2 spike epitope-specific TCR clones were mostly from EM cells, possibly related to formation of immunological memory post vaccination (Figure S9D). There was an increase in spike epitope-specific T cells detected from 1 month post D2 to 1 month post D3 in 6 of 11 individuals under 70 years of age, but only in 3 of 10 individuals from the ≥70-year-old age group (Figure 6B). Moreover, the <70-year-old cohort showed a larger increase in spike epitope-specific effector TCR clones following mRNA booster compared with the ≥70-year-old group (Figure 6C). This suggests that younger individuals mount a stronger and more diverse response following the mRNA booster vaccine. Of note, we identified one spike-specific TCR clone with identical α- and β-CDR3 sequences shared across two unrelated subjects, strongly suggesting that our method enables identification of TCR clones that emerge after vaccination (Figure S9E). In one individual (AZ-7, ≥70 years), over 30 cells from this clone were detected, with a large increase following D3, and may relate to expansion of an existing memory T cell clone following the mRNA booster. Altogether, our analysis suggests that despite increased activation signatures in effector T cells following an mRNA booster vaccine (Figure 5), older individuals exhibit muted SARS-CoV-2-specific T cell responses following the mRNA booster.

To investigate the functional implications of age- and vaccine-dose-associated differences in antigen-specific T cell responses, we measured IFN-γ and interleukin-2 (IL-2) T cell responses in PBMCs using a Fluorospot assay. PBMCs were stimulated with overlapping peptide pools derived from the D614G SARS-CoV-2 spike, and the IFN-γ and IL-2 responses were measured. There was a significant increase in IFN-γ and IL-2 responses following the mRNA booster compared with 6 months post second dose of AZD1222 (p < 0.0281 and p < 0.0291 for IFN-γ and IL-2, respectively; Figure 6D). However, this difference was driven by a robust increase in T cell responses in the <70-year-old age group, while in the ≥70-year-old age group no booster dose-associated augmentation in IFN-γ and IL-2 T cell responses was evident following the third dose of vaccine (Figures 6E, 6F, and S10). *Il2* transcripts were typically below the limit of detection in our scRNA-seq data, but among CD4 T cells some expression of *IFNG* was observed, which was greater in cells from the <70-year-old group than in the ≥70-year-old group, as was expression of *IFNGR1* in both effector CD4 and CD8 T cells (Figures S9H and S9I). These data indicate that T cell immunity conferred by AZD1222 persists, and boosting with an mRNA-based vaccine enhances responses. However, the impact of the booster, particularly for IL-2 responses, is diminished in the elderly.

### Transcriptional changes in NK cells and myeloid cells evident after mRNA booster

**Figure 7:**
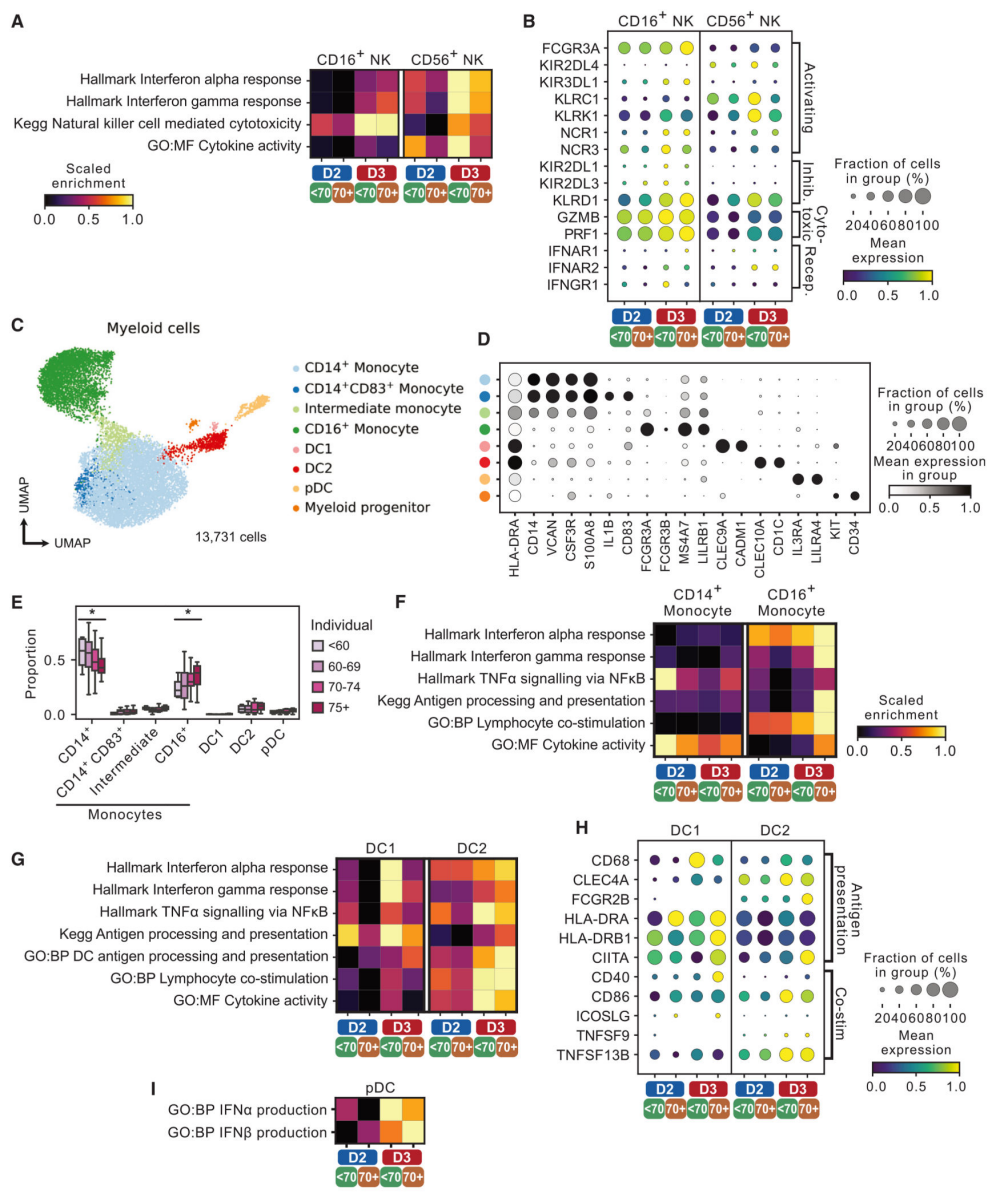
Age-associated changes in circulating NK and myeloid cells following AZD1222 and an mRNA booster (A) Heatmap showing gene set expression in NK cell subsets in <70-year-old and ≥70-year-old individuals 1 month post dose 2 AZD1222 (D2) and 1 month post mRNA booster (D3). (B) Selected differentially expressed genes driving differences in (A). (C and D) UMAP of subsetted myeloid cells (C) annotated by canonical marker gene expression (D). (E) Proportions of myeloid cell subsets 1 month post dose 2 AZD1222 (left) and 1 month post mRNA booster (right) in individual individuals in different age groups. Significance testing using Kruskal-Wallis one-way test. (F) Heatmap showing gene set expression in monocytes in <70-year-old and ≥70-year-old individuals 1 month post dose 2 AZD1222 (D2) and 1 month post mRNA booster (D3). (G) Heatmap showing gene set expression in conventional DCs in <70-year-old and ≥70-year-old individuals 1 month post dose 2 AZD1222 (D2) and 1 month post mRNA booster (D3). (H) Selected differentially expressed genes driving differences in (G). (I) Heatmap showing gene set expression in conventional DCs in <70-year-old and ≥70-year-old individuals 1 month post dose 2 AZD1222 (D2) and 1 month post mRNA booster (D3).

Finally, we interrogated the single-cell transcriptomes of the NK and myeloid cells captured in our scRNA-seq dataset. Circulating NK cells are composed of two major subsets, a CD16^+^ subset with marked cytotoxic capacity and a CD56^bright^, CD16^−^ subset associated with reduced cytotoxicity and prominent cytokine production, particularly T helper 1 cytokines such as IFN-γ.^46^ In the CD16^+^ NK cells, the expression of cytotoxicity-associated genes, including *GZMB* and *PRF1*, was higher at 1 month post mRNA vaccine booster (D3) compared with 1 month post dose 2 of AZD1222 (Figures 7A and 7B). *FCGR3A* expression encoding CD16, the IgG receptor required for NK cell antibody-dependent cellular cytotoxicity, was also higher post D3, particularly in the ≥70-year-old cohort (Figure 7B), potentially augmenting the antiviral effects of the antibodies generated in the cohort. In the CD56^+^CD16^−^ NK cell subset, “interferon alpha response” and “interferon gamma response” gene sets were more highly expressed post D3 compared with post D2, but at both time points expression was greater in the <70-year-old compared with the ≥70-year-old group (Figure 7A).

When considering the myeloid cells in isolation, CD14^+^ classical monocytes and CD16^+^ non-classical monocytes were the major subsets represented, with CD1c^+^ cDCs and pDCs the next largest populations (Figures 7C and 7D). The proportional representation of CD14^+^ monocytes decreased with age, with a corresponding increase in CD16^+^ monocytes with age (Figure 7E), in line with previous descriptions.^47^ The activating effect of the mRNA booster on this subset was particularly remarkable in the ≥70-year-old age group, which showed greater expression of “interferon alpha response,” “interferon gamma response,” “antigen processing and presentation,” and “lymphocyte co-stimulation” gene sets than that observed in the <70-year-old cohort (Figure S9). cDCs showed higher expression of “antigen processing and presentation” and “lymphocyte co-stimulation” gene sets post mRNA booster (D3) compared with post dose 2 of AZD1222 (D2), the latter particularly marked in CD1c^+^ DCs, including *CD86* and *TNFSF13B* (encoding BAFF) (Figures 7G and 7H). In pDCs, which help to control coronavirus infections via type I IFN production, there was also higher expression of “interferon alpha and beta production” gene sets post D3 compared with post D2 (Figure 7I). These data suggest that circulating pDCs are primed to respond more vigorously to viral challenge following an mRNA booster vaccine, including in elderly individuals.

## Discussion

Long-term vaccine-elicited immunity is important for protection against SARS-CoV-2 variants and can be measured by circulating binding and neutralizing antibodies, spike-specific T cell immunity, and spike-specific B cell responses.^18,48^ Neutralizing antibody levels wane over time, with a significant decrease seen 6 months after the second dose.^49,50^ In contrast, T cell immunity is longer lived and may confer durable protection, even as new variants emerge. Studies showed that the T cell response remained robust over a 6-month period, even to Omicron BA.1.^21,50,51^ Importantly, the elderly demonstrated lower neutralization titers and lower CD4 T cell IL-2 secretory responses to spike following mRNA vaccination.^26^

Compared with mRNA primary-course vaccination, two-dose AZD1222 vaccine has been shown to confer poorer protection against infection with variants of concern including Beta^52^ and Delta, with breakthrough cases emerging^8,29,53^ even when peak antibody titers are expected. With titers of neutralizing antibodies waning in the general population after mRNA or adenovirus vectored vaccine primary course,^54,55^ an mRNA booster was recommended based on early studies with mRNA as the third vaccine dose; previous studies^28,56–59^ showed that heterologous vaccination in individuals primed with AZD1222, AD26.-COV2.S, and boosted with an mRNA-based vaccine or homologous vaccination with BNT162b2, enhanced immune responses as determined by measurement of neutralizing antibodies and T cell responses. Additionally, the booster vaccine dose aided seroconversion in immunosuppressed individuals.^60^ However, few *in vitro* data exist regarding boosting in the elderly population, in contrast to epidemiological data^28,61,62^; this lack of data is particularly evident for heterologous prime-boost approaches.^56^ However, a recent meta-analysis did indicate greater protection from hospitalization for those receiving three BNT162b2 doses versus two doses of AZD1222 followed by BNT162b2 booster.^63^

Primary-course AZD1222 vaccine was approved after BNT162b2 in the UK and therefore given to younger individuals between the ages of 40 and 75 years. Following early data on boosting of immune responses after mRNA third dose,^28,58^ mRNA-based vaccines were offered as a booster vaccine 6 months after primary two-dose courses of either AZD1222 or BNT162b2. In our cohort of 36 individuals, 13 whom were 70 years or older, we assessed binding and neutralizing responses as well as T cell and B cell responses to vaccination over time. Significant waning of neutralizing antibodies was observed across all individuals 6 months post second dose, but 1 month after mRNA-based booster vaccination the titers increased significantly to levels that were also significantly higher than those seen 1 month after the second dose of AZD1222. Interestingly, no differences were observed between age groups for doses 1 and 2. However, following booster vaccination, the ≥70-year-old group did not respond as well as the under-70 group. However, while age-related differences were observed in the neutralization, total spike IgG levels showed no association with age. This pointed toward differences in neutralization potency and possibly breadth, rather than quantity, of spike-specific antibody. We also observed suboptimal boosting of spike-specific T cell responses in the elderly after dose 3 that was most marked for the IL-2 response, which we previously showed was largely derived from CD4 cells. Spike-specific T cell expansion was also impaired post dose 3 in the elderly. This was accompanied by lower levels of T cell activation as well as lower innate immune activation gene signatures following the priming doses of AZD1222 compared with dose 3 BNT162b2.

While variable region binding to antigen is important for neutralization, Fc-mediated IgG effector functions such as NK cell antibody-dependent cellular cytotoxicity require binding to cell-surface FcγRs. In this regard, our scRNA-seq analysis showed that *FCGR3A* (CD16) expression on NK cells was higher post D3 in the ≥70-year-old cohort compared with those <70 years old, potentially acting to compensate for the effect of reduced viral antibody neutralization in this cohort. CD16^+^ monocytes in the ≥70-year-old cohort also showed a greater enrichment of a variety of activation gene signatures post D3 compared with the <70-year-old group.

A previous analysis of responses to a second dose of mRNA vaccine (following primary mRNA vaccine dose) found that early monocyte activation correlated with the development of SARS-CoV-2 neutralizing antibodies and CD8 T cell IFN-γ responses.^64^ Altogether, our scRNA-seq analysis suggests that even a month after the booster mRNA vaccine, there is evidence of ongoing transcriptional activation of monocytes, pDCs, and cDCs, with expression of several genes that may promote T and B cell activation. In contrast to adaptive immune cells, myeloid cells do not exhibit classical immunological memory. Therefore, the enhanced myeloid cell activation observed in response to the mRNA vaccine relative to dose 2 of AZD1222 likely reflects a vaccine-intrinsic feature.

Phenotyping RBD-specific B cells from 1 month post boost revealed a distinct population of IgD^−^RBD^+^ age-associated atypical memory B cells, which was present at a higher frequency in older individuals than in younger participants. The literature surrounding atypical memory B cells describes various roles in humans, although these different functions may be context dependent.^65^ Initially, B cells with this phenotype were characterized as exhausted or hyporesponsive memory B cells that formed after infection or in autoimmune disease.^66–68^ Additionally, there was an accumulation of atypical memory B cells in older individuals, suggesting that biological changes that occur with age can favor skewing of the memory B cell pool toward an atypical B cell fate.^34,69^ The formation of atypical memory B cells can be supported by IL-21 and IFN-γ and be inhibited by IL-4^34^; therefore, these cells may emerge as a natural consequence of the increased inflammation that is present in older people. We have previously shown that hemagglutinin-specific circulating T follicular helper cells that are induced by vaccination have an enhanced IFN-γ gene signature in older donors,^41^ indicating that atypical B cell promoting conditions exist in older people upon vaccination. Indeed, our scRNA-seq analysis demonstrated a greater enrichment of “interferon gamma response” genes across all B cell subsets post D3 in the ≥70-year-old age group compared with those <70 years old, suggesting that this may underpin the age-associated expansion in atypical B cells in this context.

Although first described in immune pathology, it is now clear that atypical memory B cells emerge from normal B cell activation in response to vaccination.^65,69–73^ Most studies suggest that the majority of atypical B cells are non-GC-derived.^69,74,75^ We have previously described that AZD1222 elicits a diminished GC response in aged mice compared with younger animals^76^ and that this vaccine can stimulate atypical B cell generation from both GC-derived and non-GC-derived pathways. The poor GC observed in older individuals may skew B cell differentiation to the extrafollicular pathway, enhancing the output of typical B cells, as has been reported in autoimmunity. Our data highlight that vaccine format can overcome this age-dependent accrual of vaccine-specific atypical B cells and represents a tractable approach to supporting immunity in older persons.

### Limitations of the study

Limitations include relatively modest sample size, sampling of peripheral blood to measure vaccine-induced immune responses, and lack of clinical data on protection from subsequent SARS-CoV-2 infection and severity. Multivariant analysis for co-morbidities was not possible, due to the small sample size.

Going forward, it will be important to understand the dynamics of waning in elderly individuals, as well as the impact of subsequent doses and differences by age. Such studies are increasingly challenging, due to the heterogeneity in time intervals between vaccine doses and natural infection. Nonetheless, the elderly remains a key target population for maximizing protective vaccine responses as they are still disproportionately likely to have poor health outcomes after SARS-CoV-2 infection, warranting continued comprehensive assessment.

## Star⋆Methods

### Key Resources Table

**Table:** 

REAGENT or RESOURCE	SOURCE	IDENTIFIER
Antibodies		
Fixable Far Red Dead Cell Stain Kit	Thermo Fisher Scientific	L10120
BD Horizon™ BUV395 Mouse Anti-Human CD27	BD	AB_2744349
CD57 Antibody (TB01) [Alexa Fluor® 350]	Novus Biologicals	AB_2909528
BD Horizon™ BUV496 Mouse Anti-Human CD4	BD	AB_2813886
BD OptiBuild™ BUV563 Mouse Anti-Human FCRL5 (CD307e)	BD	AB_2873900
BDOptiBuild™ BUV615 Mouse Anti-Human CD19	BD	AB_2875287
BD Horizon™ BUV661 Mouse Anti-Human CD11c	BD	AB_2870241
BD Horizon™ BUV737 Mouse Anti-Human CD10	BD	AB_2871160
BD OptiBuild™ BUV805 Mouse Anti-Human CD38	BD	AB_2871359
Brilliant Violet 421™ anti-human/mouse/rat CD278 (ICOS) Antibody	BioLegend	AB_2562545
T-bet Monoclonal Antibody (eBio4B10 (4B10)), eFluor™ 450, eBioscience™	Thermo Fisher Scientific	AB_2784727
BD OptiBuild™ BV480 Mouse Anti-Human CD21	BD	AB_2743893
BDOptiBuild™ BV510 Mouse Anti-Human TCR γδ	BD	AB_2739932
Mouse anti Human CD45RA:StarBright Violet 570	BioRad	AB_871980
BD OptiBuild™ BV650 Mouse Anti-Human CD183	BD	AB_2740303
BD Horizon™ BV711 Mouse Anti-GATA3	BD	AB_2739242
BD OptiBuild™ BV750 Mouse Anti-Human CD279 (PD-1)	BD	AB_2872125
BD Horizon™ BV786 Mouse Anti-Human HLA-DR	BD	AB_2738559
BD Horizon™ BB515 Rat Anti-Human CXCR5 (CD185)	BD	AB_2738871
IgM Antibody (IM373) [Alexa Fluor® 532]	Novus Biologicals	AB_2909529
Spark Blue™ 574 anti-human CD3 Antibody	BioLegend	AB_2904329
CD14 Monoclonal Antibody (TuK4), PerCP	Thermo Fisher Scientific	AB_10374157
CD196 (CCR6) Monoclonal Antibody (R6H1), PerCP-eFluor™ 710, eBioscience™	Thermo Fisher Scientific	AB_10597900
BD OptiBuild™ BB700 Mouse Anti-Human CD71	BD	AB_2743458
BD BB790 IRF4 antibody	BD custom conjugation	N/A
Spark YG™ 593 anti-mouse/humanCD11b Antibody	BioLegend	AB_2892261
Alexa Fluor® 594 anti-human CD44 Antibody	BioLegend	AB_2860987
PE/Dazzle™ 594 anti-human CD25 Antibody	BioLegend	AB_2563562
CD24 Monoclonal Antibody (SN3), PE- Alexa Fluor™ 610	Thermo Fisher Scientific	AB_1468089
PE/Cyanine5 anti-human CD184 (CXCR4) Antibody	BioLegend	AB_314614
FOXP3 Monoclonal Antibody, PE- Cyanine5, eBioscience™	Thermo Fisher Scientific	AB_891552
ROR gamma (t) Monoclonal Antibody (B2D), PE-Cyanine7, eBioscience™	Thermo Fisher Scientific	AB_2784671
PE/Fire™ 810 anti-human CD197 (CCR7) Antibody	BioLegend	AB_2894572
Spark NIR™ 685 anti-human CD20 Antibody	BioLegend	AB_2860775
Ki-67 Monoclonal Antibody (SolA15), Alexa Fluor™ 700, eBioscience™	Thermo Fisher Scientific	AB_2637480
APC/Fire™ 750 anti-human IgD Antibody	BioLegend	AB_2616988
APC/Fire™ 810 anti-human CD8 Antibody	BioLegend	AB_2860890
Bacterial and virus strains		
DH5α Competent Cells	Thermo Fisher Scientific	Cat#: 18265017
Biological samples		
SARS-CoV-2 vaccinated human sera and plasma	ARIA study (2014-2014 at NIHR BioResource Center, Cambridge UK	N/A
Chemicals, peptides, and recombinant proteins		
FuGENE® HD Transfection Reagent	Promega	E2312
PepTivator® CEF MHC Class I Plus	Miltenyi Biotec	130-098-426
Recombinant SARS-CoV-2 Spike-Prot (HEK)	Miltenyi-Biotec	130-127-681
Brilliant Violet 605™ Streptavidin	BioLegend	405229
PE Streptavidin	BioLegend	405204
APC Streptavidin	BioLegend	405207
Alexa Fluor® 647 Streptavidin	BioLegend	405237
ViaKrome 808 Fixable Viability Dye	Beckman Coulter	C36628
FoxP3/Transcription Factor Staining buffer	eBioscience	Cat#00-5323-00
Permeabilization buffer	eBioscience	Cat#00-8333-56
Biotin ≥99% (HPLC), lyophilized powder	Sigma-Aldrich	CAS Number: 58-85-5
Ni-NTA Agarose	Qiagen	Cat#: 30210
Normal Rat Serum	Sigma-Aldrich	Cat#: R9759
*p*-Nitrophenyl Phosphate Substrate Buffer	Sigma-Aldrich	Cat#: 487664
Critical commercial assays		
Bright-Glo	Promega	Cat#E2650
FluoroSpot^FLEX^ IFN-γ and IL-2	Mabtech	N/A
Luminex® Assay	R&D Systems	N/A
10x Chromium GEM Single Cell V(D)J 5’ kit	10X Genomics	N/A
Deposited data		
EGAS00001007385	N/A	N/A
Experimental models: Cell lines		
HEK239T	ATCC	Cat#CRL-3216
ACE2 – HeLa Recombinant Cell Line	Kind gift from Dr. James Voss, SCRIPPS	N/A
Experimental models: Organisms/strains		
Human peripheral blood samples from SARS-CoV-2 mRNA and AdV vaccine recipients	Collected at NIHR BioResource Center, Cambridge	N/A
Human serum samples from SARS-CoV-2 mRNA vaccine recipients	Collected at NIHR BioResource Center, Cambridge	N/A
Human peripheral blood samples from SARS-CoV-2 mRNA vaccine recipients	Collected at COVID-19 PROTECT study, Singapore	N/A
Human Serum	ThermoFisher	Catalog #R0001-0A
Recombinant DNA		
Plasmid: SARS-CoV-2 spike D614-FLAG	Biobasic	N/A
Plasmid: p8.91	This paper	N/A
Plasmid: CSFLW	This paper	N/A
Plasmid: pcDNA3.1	Thermo Scientific, Invitrogen	Cat#V66020
Plasmid: human ACE2 receptor	Biobasic	N/A
Plasmid: TMPRSS2	Biobasic	N/A
Plasmid: BirA	This paper	N/A
Plasmid: RBD-avi-His	This paper	N/A
SARS-CoV-2 Spike	BioBasic	Wuhan strain QHR63290.2
Software and algorithms		
Prism	GraphPad	https://www.graphpad.com
CellRanger v7.0	10X Genomics	N/A
SoupOrCell v2.0	Open source (pip)	N/A
Scanpy v1.9.3	Open source (pip)	N/A
BBKNN v1	Open source (pip)	N/A
Dandelion v0.2	Open source (pip)	N/A
FlowJo	Treestar	https://www.flowjo.com/
GraphPad Prism	GraphPad	https://www.graphpad.com/
R	RStudio	https://www.r-project.org

### Resource Availability

#### Lead contact

Further information should be directed to and will be fulfilled by the lead contact, Ravindra K. Gupta (rkg20@cam.ac.uk).

#### Materials availability

This study did not generate new unique reagents.

### Experimental Model and Study Participant Details

The study was primarily a laboratory-based study using pseudotyped virus (PV) with mutations generates by site directed mutagenesis. Sensitivity to antibodies in serum was tested using convalescent sera from recovered individuals, along with B cell phenotyping, and single cell RNA sequencing, collected as part of the Cambridge NIHR Bioresource. We also performed phylogenetic analyses of data available publicly in GISAID.

Experiments were performed on peripheral blood mononuclear cells (PBMCs) and serum that were collected from individuals and cryopreserved. These individuals were vaccinated with either two doses of AZD1222 and an mRNA booster or three doses of an mRNA vaccine. Twenty-three women and thirteen men were included in the study with a median age of 66 years of age for the women and 73 years of age for the men.

From the cohort recruited in Singapore, all vaccinated participants received two doses of the Pfizer/BioNTech BNT162b2 mRNA vaccine at 21 days apart. Three plasma samples were collected from each participant: three months after the first dose (i.e., peak response); and six months after the first dose. In addition, plasma sample from a fifth timepoint at one to three months after the booster dose (i.e., third dose) were collected. The young cohort consisted of ten women and ten men in the young cohort and thirteen women (median age of twenty-nine years of age) and twenty-five men (median age of thirty-two years of age). The elderly cohort consisted of thirteen women (median age of seventy and a half years of age) and twenty-five men (median age sixty-nine years of age)

#### Ethical approval

The study was approved by the East of England – Cambridge Central Research Ethics Committee (17/EE/0025). PBMC from unexposed volunteers previously recruited by the NIHR BioResource Center Cambridge through the ARIA study (2014–2016), with ethical approval from the Cambridge Human Biology Research Ethics Committee (HBREC.2014.07) and currently North of Scotland Research Ethics Committee 1 (NS/17/0110).

The vaccinated participants were recruited under the COVID-19 PROTECT study (2012/00917) in Singapore. All participants provided written informed consent in accordance with the Declaration of Helsinki for Human Research. Ethics committee of National Healthcare Group (NHG) Domain Specific Review Board (DSRB) Singapore gave ethical approval for this work.

### Method Details

#### Generation of Mutants and pseudotyped viruses

Wild-type (WT) bearing 614G, B.1.617.2 (Delta), and B.1.1.529 (Omicron BA.1) pseudotyped viruses were generated as previously described.^31^ In brief amino acid substitutions were introduced into the D614G pCNA_SARS-CoV-2_S plasmids as previously described.^3^ The pseudoviruses were generated in a triple plasmid transfection system whereby the Spike expressing plasmid along with a lentviral packaging vector-p8.9 and luciferase expression vector-psCSFLW where transfected into 293T cells with Fugene HD transfection reagent (Promega). The viruses were harvested after 48 h and stored at −80°C. TCID50 was determined by titration of the viruses on 293Ts expressing ACE-2 and TMPRSS2.

#### Neutralization assays

Virus neutralization assays were run using HeLa expressing ACE2 cells using SARS-CoV-2 Spike pseudotyped virus expressing luciferase. Pseudotyped virus was incubated with serially diluted heat inactivated human serum samples or sera from vaccinees in duplicate for 1h at 37°C. Cell only and virus and cell only controls were included. After an hour, HeLa ACE2 cells were added to each well. Following 48h of incubation at 5% CO_2_ and 37°C, luminescence was measured using the BrightGlo Luciferase Assay System (Promega, UK). Neutralization was calculated relative to the virus and cell only controls. Data was analyzed in GraphPad Prism where 50% neutralization (ID50) values were calculated and the limit of detection for neutralization was set at an ID50 of 20. Within each group, the ID50 values were summarized a geometric mean titer (GMT). Statistical comparisons between groups were made using either the Wilcoxon ranked sign test or the Mann-Whitney test.

#### SARS-CoV-2 serology by multiplex particle-based flow cytometry (Luminex)

Recombinant SARS-CoV-2 N, S and RBD were covalently coupled to distinct carboxylated bead sets (Luminex; Netherlands) to form a 3-plex and analyzed as previously described.^31^ Specific binding was reported as mean fluorescence intensities (MFI).

#### Spectral flow cytometry

Fluorescent RBD and Spike specific probes were generated and used in spectral flow cytometry panels as previously reported.^77^ UMAP analysis of flow cytometry data was using performed R (version 4.1.2) using code that has previously been described.^78^

#### IFNγ and IL-2 FLUOROSPOT T cell assays

Peripheral blood mononuclear cells (PBMC) were isolated from the heparinized blood samples using Histopaque-1077 (Sigma-Aldrich) and SepMate-50 tubes (Stemcell Technologies). Frozen PBMCs were rapidly thawed and diluted into 10mL of TexMACS media (Miltenyi Biotech), centrifuged and resuspended in 10mL of fresh media with 10U/ml DNase (Benzonase, Merck-Millipore via Sigma-Aldrich), PBMCs were then incubated at 37°C for 1h, followed by centrifugation and resuspension in fresh media supplemented with 5% Human AB serum (Sigma Aldrich) before being counted. PBMCs were stained with 2ul of LIVE/DEAD Fixable Far Red Dead Cell Stain Kit (Thermo Fisher Scientific) and live PBMC enumerated on the BD Accuri C6 flow cytometer.

1.0 to 2.5 x 10^5^ PBMCs were incubated in pre-coated FluoroSpot^FLEX^ plates (anti IFNγ and IL-2 capture antibodies Mabtech AB, Nacka Strand, Sweden)) in duplicate with either peptide mixes specific for Wuhan-1(QHD43416.1) Spike SARS-CoV-2 protein (Miltenyi Biotech) or a mixture of peptides specific for Cytomegalovirus, Epstein-Barr virus and Influenza virus (CEF+) (final peptide concentration 1 μg/ml/peptide, Miltenyi Biotech) in addition to an unstimulated (media only) and positive control mix (containing anti-CD3 (Mabtech AB) and Staphylococcus Enterotoxin B (SEB), (Sigma Aldrich) at 37°C in a humidified CO_2_ atmosphere for 42 h. The cells and medium were then decanted from the plate and the assay developed following the manufacturer’s instructions. Developed plates were read using an AID iSpot reader (Oxford Biosystems, Oxford, UK) and counted using AID EliSpot v7 software (Autoimmun Diagnostika GmbH, Strasberg, Germany). Peptide specific frequencies were calculated by subtracting for background cytokine specific spots (unstimulated control) and expressed as SFU/Million PBMC.

#### Sample processing, library preparation, and sequencing

PBMC samples were removed from −80 storage and defrosted by gradual addition and removal of ice-cold PBS, resuspending the frozen cells to a final volume of 40 mL while keeping the samples on wet ice throughout defrosting. The cells were centrifuged at 400g for 5 min. The supernatant was discarded, and cells were re-suspended in a small volume of PBS with CaCl_2_, as required for enrichment of live cells, using EasySep (STEMCELL technologies) dead cell removal kit, following the manufacturer’s instructions. Following this, cells were centrifuged as before and counted. Two or three samples from distinct individuals were pooled (i.e., genotype multiplexed) in an overlapping mixture design at equal concentrations, counted, and 1x10^5^ cells were resuspended in 100μL of PBS.

*The 10x Chromium GEM Single Cell V*(*D*)*J 5’ kit v2* (*dual index*) *with BCR and TCR* amplification was used for library preparation. Samples were loaded onto the chip following the manufacturer’s recommendations, with an aim to recover 8000 cells (for 2 samples) or 12000 cells (for 3 samples) per lane. The remainder of the 10x library preparation was carried out as per manufacturer’s instructions and the resulting libraries (GEX, TCR, BCR) sequenced using NovaSeq 6000 paired-end sequencing (Illumina) at Genewiz. BCL files were demultiplexed using Casava (Illumina) and count tables produced using CellRanger v7.0 (10x genomics).

#### Single-cell RNA-seq data and pre-processing

Genotype demultiplexing was performed using Souporcell (v2).^79^ Souporcell analyses was performed using the ‘skip_remap’ setting and a set of known donor genotypes given under the ‘common_variants’ parameter, and the k number set at the number of samples loaded per lane. The donor ID for each Souporcell genotype cluster was annotated by comparing with known genotypes from the multiplex design.^80^ ^8181^(81)[81](“bjstewart1/GenotypeMixtures: Stitches together genotype clusters from multiple Souporcell results over large single cell genomics experiments. https://github.com/bjstewart1/GenotypeMixtures.,”) Droplets containing more than one genotype according to Souporcell or with unresolved genotypes were removed. Further doublet detection was performed on the combined raw count data (10x CellRanger output) using Scrublet (v0.2.3).^81^ Following this, iterative sub-clustering was performed, the median Scrublet score for each sub-cluster was computed, and median absolute deviation scores were calculated followed by application of a one-tailed t test with Benjamin-Hochberg correction, as previously described.^82^ Cells with significantly outlier Scublet scores (corrected Pval <0.05) were regarded as probable doublets and filtered. The data was then processed using Scanpy following the standard workflow.^83^ Cells were filtered if they contained >200 or <8000 genes. Percentage mitochondrial content cut-off was set at <15%. Genes were retained if they were expressed in three or more cells. Highly variable genes were selected based on a minimum and maximum expression of >0.0125 and <3 respectively; with the minimum dispersion of genes = 0.5. TCR and BCR V(D)J genes were removed from highly variable genes. The number of PCs used for neighborhood graph construction and dimension reduction was set at 30. Batch correction was performed using bbknn using the ridge regression setting and 10x sequencing lane as the batch term.^84,85^ Clustering was performed using the Leiden algorithm.^86^ Visualization of reduced dimensions was performed with UMAP (v3.10.0) using a minimum distance of 0.3 and all other parameters according to the default settings in Scanpy.^87^ For initial clustering, differentially expressed genes were calculated using the Wilcoxon ranksum test. Finally, cell clusters expressing improbable combination of cell type markers were filtered, after manual inspection of the data. This led to a working dataset of 99,384 cells.

#### Single-cell gene expression analysis

Preliminary annotation of cell clusters was performed with CellTypist.^37^ Briefly, the ‘Covid19 immune landscape’ model was used to predict cell-types based on logistic regression classifiers, using the majority voting classifier setting. Next, clusters were manually inspected, to obtain the final annotations using a combination of canonical mRNA markers and BCR/TCR sequencing information, where available. Gaussian kernel density estimation was performed using Scanpy’s tl.embedding_density function. Compositional analysis was performed using scCODA, which applies a Bayesian model to identify cell type changes.^88^ Gene sets were obtained from the Molecular Signature Database (MSigDB v7.3) inventory.^89^ Gene signature scoring was performed with UCell, which is based on the Mann-Whitney U statistic.^90^ For patient-level comparisons, cell-level scores were averaged (mean) by sample, for each cell type. Mann-Whitney U test was applied for age comparisons or Wilcoxon signed-rank test for dose comparisons, where paired patient samples were available.

#### SARS-CoV-2 TCRseq analysis

For identification of putative paired TCR sequences with capacity to bind SARS-CoV2 spike antigen-derived epitopes, SARS-CoV-2 specific TCR CDR3 sequences were obtained from the Immune Epitope Database (IEDB) and VDJdb databases.^43,44^ First, VDJ gene calls and CDR3 amino acid sequences were analysed using the tcrdist package,^91^ implemented in Python, to obtain TCR distances for all pairwise combinations of TCRs in the repertoire. The pairwise TCR distance matrix was binarized using a numerical threshold and clustered using unsupervised Leiden clustering to yield sequence motifs with maximum intra-motif sequence similarity. Next, TCR sequences were compared to the IEDB and VDJdb databases. If an exact match in either the alpha or beta chain were found when compared to our scTCR-seq data, the TCR and all TCRs within the same motif cluster were labeled as SARS-CoV-2 specific, followed by further identification of spike epitope-specific sequences if the epitope gene name contained ‘Surface’, ‘Spike’, or ‘S’ in the databases. Altogether, this approach led to the identification of 190 putative SARS-CoV-2 spike epitope-binding single-cell TCRs.

### Quantification and Statistical Analysis

Descriptive analyses of demographic and clinical data are presented as median and interquartile range (IQR) when continuous. When categorical, these data are presented as frequency and proportion (%). Linear regression was used to model the association between age and S total IgG at each time point as well as the association between S total IgG and ID50 for the same time point. Pearson’s correlation was used to measure the relationship between the variables. Linear regression was also used to measure the association between IFN-γ and ID50. Statistical analyses were run using GraphPad Prism. UMAP analysis was performed using R (version 4.1.1) using code that has previously been described.^78^ Measurements were done in duplicate and relative luciferase units measured with a Glomax luminometer. Data were analyzed using GraphPad PRISM software (version 9.0.0). Statistical tests are described in the figure legends along n, mean, and standard deviation/error. Data were normally distributed consistent with statistical methods used.

## Supplementary Material

Supplemental information can be found online at https://doi.org/10.1016/j.celrep.2023.112991.

Supplemental information

## Data Availability

Raw anonymized data are available from the lead contact without restriction. Raw sequencing data has been deposited on the EGA genome-phenome archive, under the study ascension number EGAS00001007385. This paper does not report original code or software. All computational methods used have been referenced and are publicly available. Any additional information to reanalyze the data reported is available from the lead contact upon reasonable request.

## Highlights

- Two doses AZD1222 and BNT162b2 dose 3 show lower neutralization in older individuals
- Reduced neutralization is not related to binding antibody or spike-specific B cell frequency
- Reduced neutralization associates with circulating “atypical” B cells in the elderly
- T cell responses, especially IL-2 secretion, show age-associated impairment post dose 3

## In brief

Ferreira et al. show that older individuals vaccinated with two doses of AZD1222 and a third dose with mRNA achieve lower neutralizing antibody responses against SARS-CoV-2 compared with younger individuals and associate with spike-specific atypical B cells. Age-related differences in serum neutralization are not observed following three mRNA vaccine doses.
