# Supplementary material for: Atypical B cells and impaired SARS-CoV-2 neutralization following heterologous vaccination in the elderly
Source: Cell Rep. Author manuscript; Available in PMC 2026 Apr 17. (PMC7619014; doi:10.1016/j.celrep.2023.112991)
Supplement: Supplemental information [file EMS213161-supplement-Supplemental_information.pdf]

Cell Reports, Volume *42*

Supplemental information

Atypical B cells and impaired SARS-CoV-2 neutralization following heterologous vaccination in the elderly

Isabella A.T.M. Ferreira, Colin Y.C. Lee, William S. Foster, Adam Abdullahi, Lisa M. Dratva, Zewen Kelvin Tuong, Benjamin J. Stewart, John R. Ferdinand, Stephane M. Guillaume, Martin O.P. Potts, Marianne Perera, Benjamin A. Krishna, Ana Peñalver, Mia Cabantous, Steven A. Kemp, Lourdes Ceron-Gutierrez, Soraya Ebrahimi, The CITIID-NIHR BioResource COVID-19 Collaboration, Paul Lyons, Kenneth G.C. Smith, John Bradley, Dami A. Collier, Laura E. McCoy, Agatha van der Klaauw, James E.D. Thaventhiran, I. Sadaf Farooqi, Sarah A. Teichmann, Paul A. MacAry, Rainer Doffinger, Mark R. Wills, Michelle A. Linterman, Menna R. Clatworthy, and Ravindra K. Gupta

Supp 1

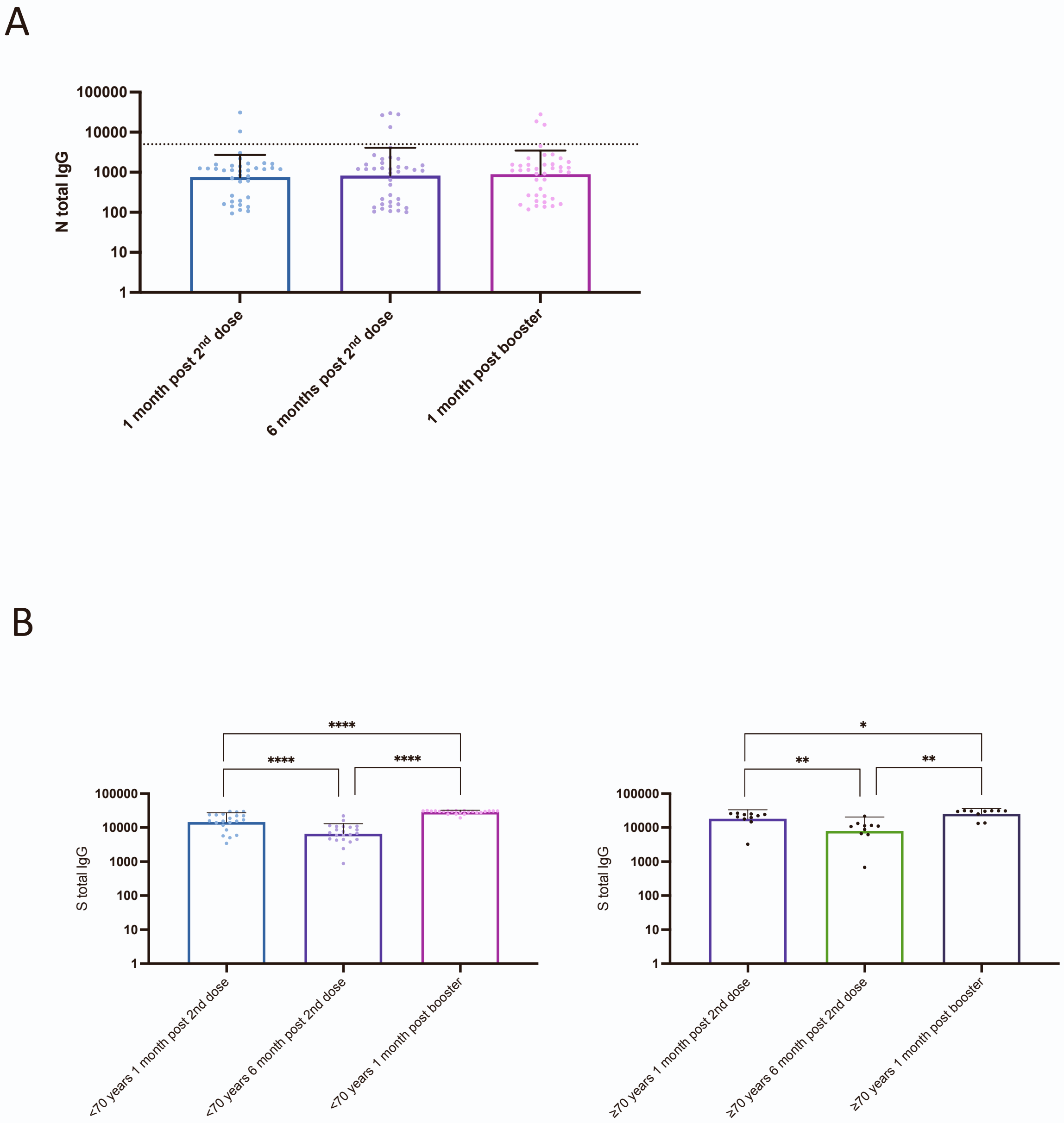

Supplementary Figure 1. Study participant characteristic data and S and N antibody responses to two doses of AZD1222 and an mRNA booster.
(A) N total IgG from study participants. Those with an MFI of 5000 or above were excluded from the study. (B) S total IgG measured from study participants at each time point stratified by those younger than 70 years and those 70 years and older. Wilcoxon matched-pairs signed ranked test was used. p * < 0.05, p ** < 0.01, p *** < 0.001, p **** < 0.0001.

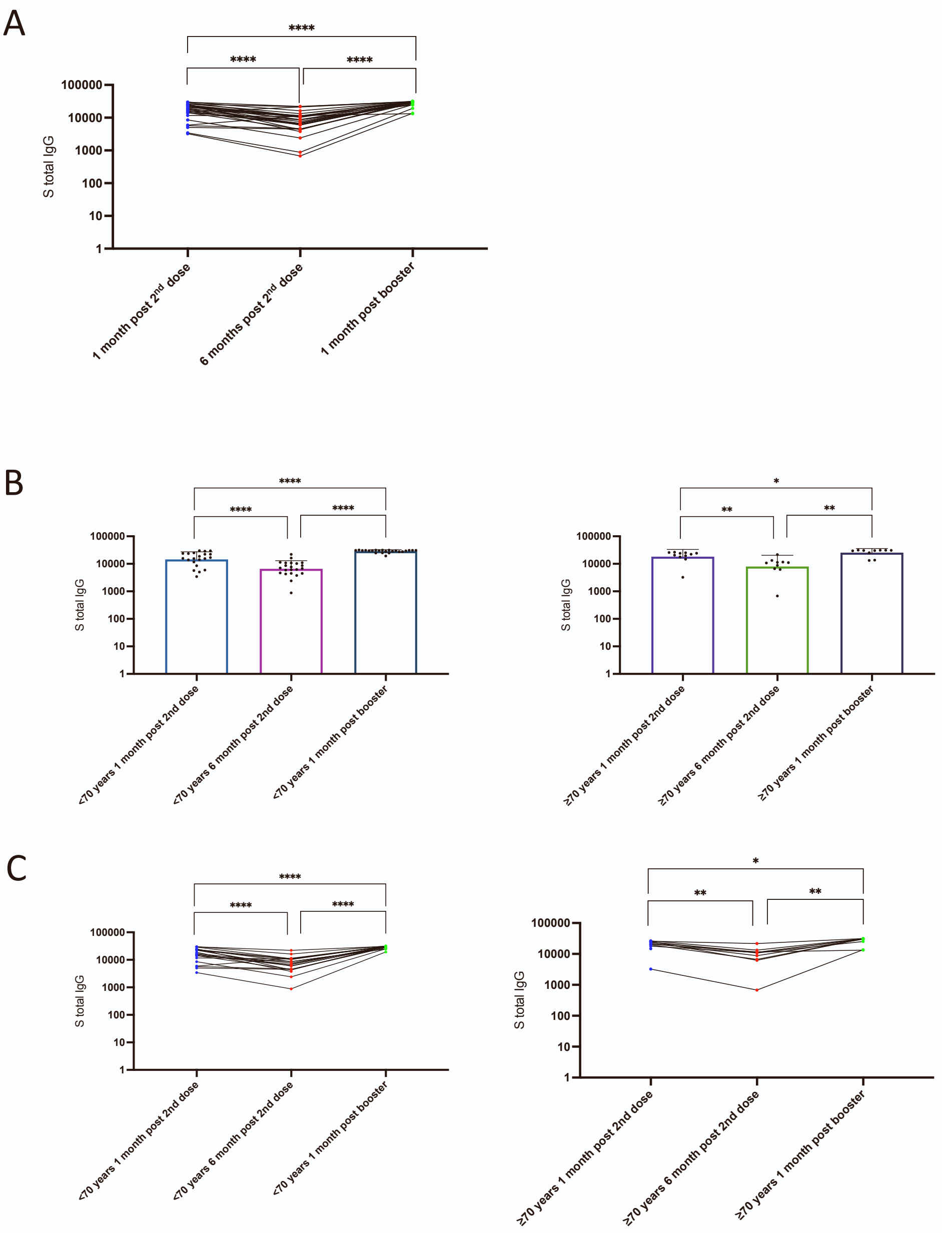

Supplementary Figure 2: S antibody responses elicited by AZD1222 vaccine and mRNA booster

(A) Linked S antibody responses from longitudinal time points across individuals. (B) S antibody responses in the <70 population and the >70 population. Wilcoxon matched-pairs signed ranked test was used. (C) Linked S antibody responses in the <70 population and the >70 population. Wilcoxon matched-pairs signed ranked test was used. p * < 0.05, p ** < 0.01, p **** < 0.0001.

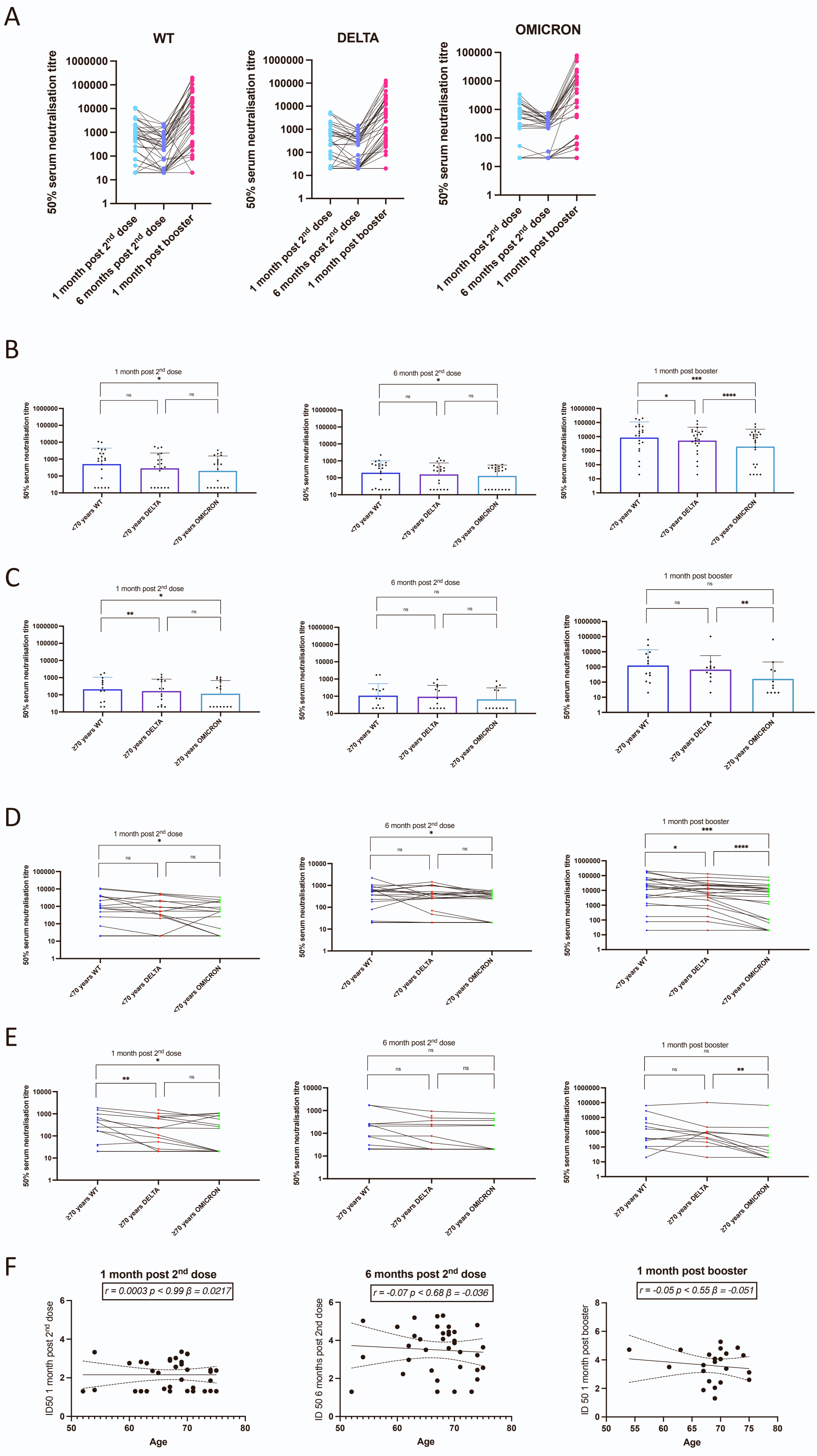
A
WT
DELTA
OMICRON
50% serum neutralisation titre
1 month post 2nd dose
6 months post 2nd dose
1 month post booster
B
1 month post 2nd dose
6 month post 2nd dose
1 month post booster
<70 years WT
<70 years DELTA
<70 years OMICRON
C
≥70 years WT
≥70 years DELTA
≥70 years OMICRON
D
E
F
1 month post 2nd dose
r = 0.0003 p < 0.99 β = 0.0217
ID50 1 month post 2nd dose
Age
6 months post 2nd dose
r = -0.07 p < 0.68 β = -0.036
ID 50 6 months post 2nd dose
1 month post booster
r = -0.05 p < 0.55 β = -0.051
ID 50 1 month post booster

Supplementary Figure 3: Longitudinal neutralizing plasma antibody titers against Wu-1 D614G WT, Delta, and Omicron variants from AZD1222 vaccinated individuals boosted with an mRNA-based vaccine.

(A) Linkage of neutralising antibodies from longitudinal time points across individuals in response to different variants. (B) Neutralising antibody titers from the <70 group at each time point against WT, Delta, and Omicron. Wilcoxon matched-pairs signed ranked test was used. (C) Neutralising antibody titers from the >70 group at each time point against WT, Delta, and Omicron. Wilcoxon matched-pairs signed ranked test was used. (D) Linkage of neutralising antibodies from longitudinal time points from the <70 group against WT, Delta, and Omicron. Wilcoxon matched-pairs signed ranked test was used. (E) Linkage of neutralising antibodies from longitudinal time points from the >70 group against WT, Delta, and Omicron. Wilcoxon matched-pairs signed ranked test was used.

Supplementary figure 4

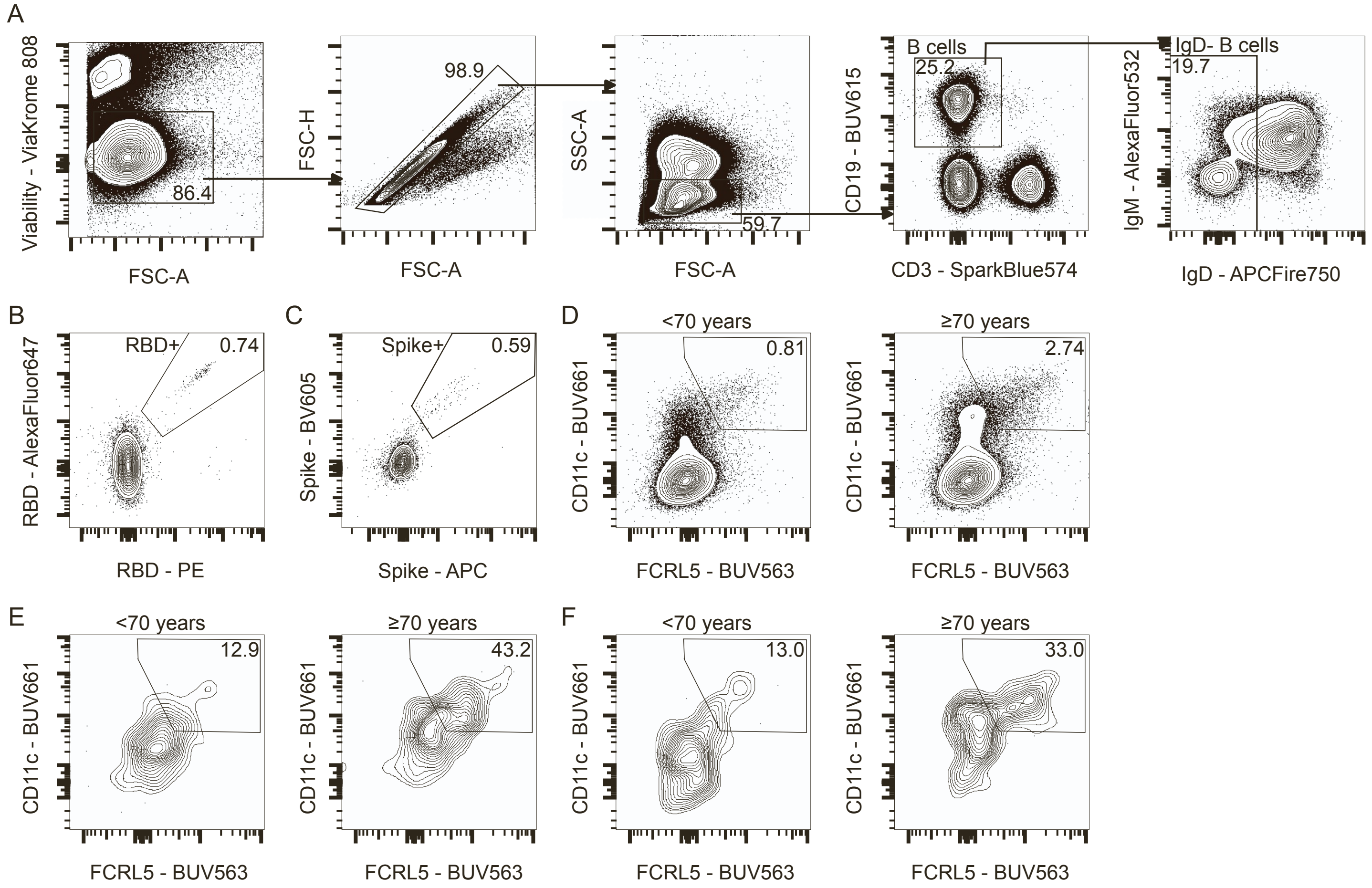

Supplementary figure 4: Flow cytometry gating for spike-specific B cell phenotyping. (A) Flow cytometry gating strategy used to define IgD- B cells. (B) Flow cytometry gating of RBD+ cells (pre-gated on IgD- B cells as in (A)). (C) Flow cytometry gating of Spike+ cells (pre-gated on IgD- B cells as in (A)). (D) Flow cytometry gating of CD11c+ FCRL5+ cells (pre-gated on B cells as in (A)). (E) Flow cytometry gating of CD11c+ FCRL5+ cells (pre-gated on RBD+ IgD- B cells as in (B)). (F) Flow cytometry gating of CD11c+ FCRL5+ cells (pre-gated on Spike+ IgD- B cells as in (C)).

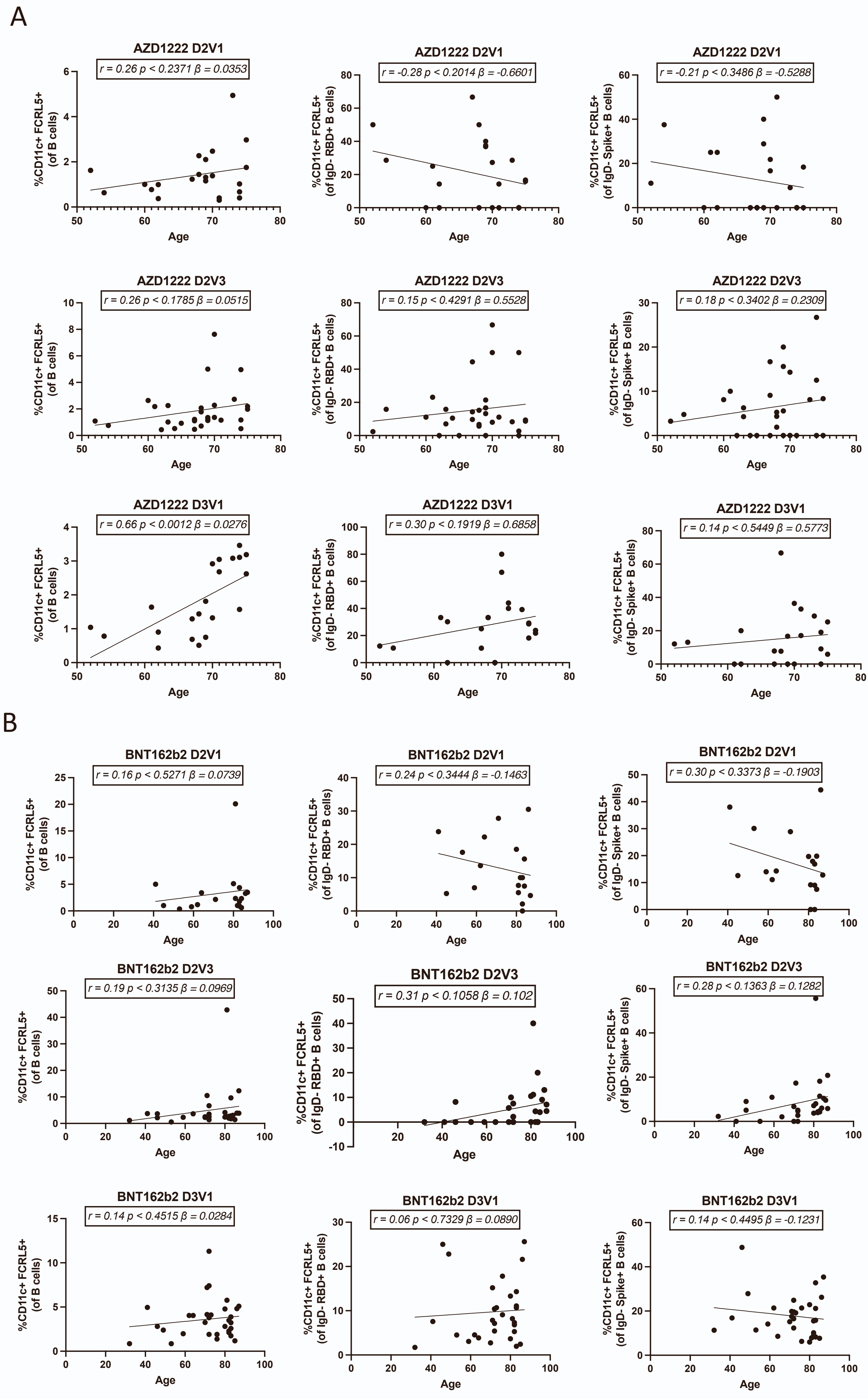

Supplementary figure 5. The relationship between age and frequency of spike specific atypical B cells following (A) primary vaccination series of two doses of AZD1222 and following booster dose with BNT162b2 or (B) three doses of BNT162b2

Correlation between atypical B cell frequency and age at each sampling time point, D2V1 – one month post dose 2 of vaccine, D2V3 (6 months post dose 2 of vaccine) and D3V1 (1 month post dose 3 of vaccine).

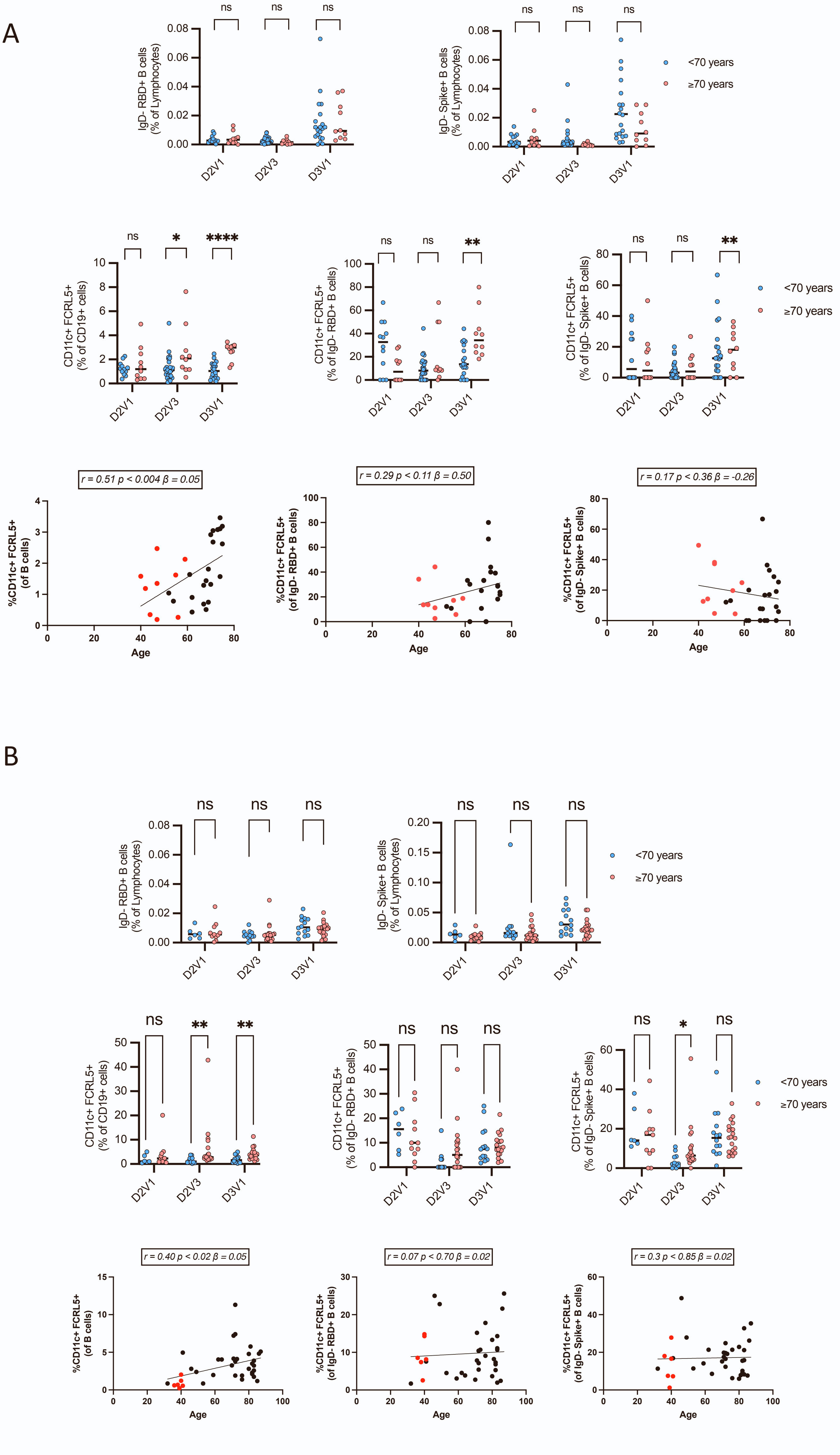

Supplementary Figure 6: Atypical B cell expression in samples from vaccinees including younger cohort in **A**. individuals vaccinated with 2 doses of AZD1222 and an mRNA booster and **B**. individuals vaccinated with 3 doses of BNT162b2. NS is non-significant, * p < 0.05, ** p < 0.01, ***p<0.001, **** p<0.0001

Supplementary figure 7

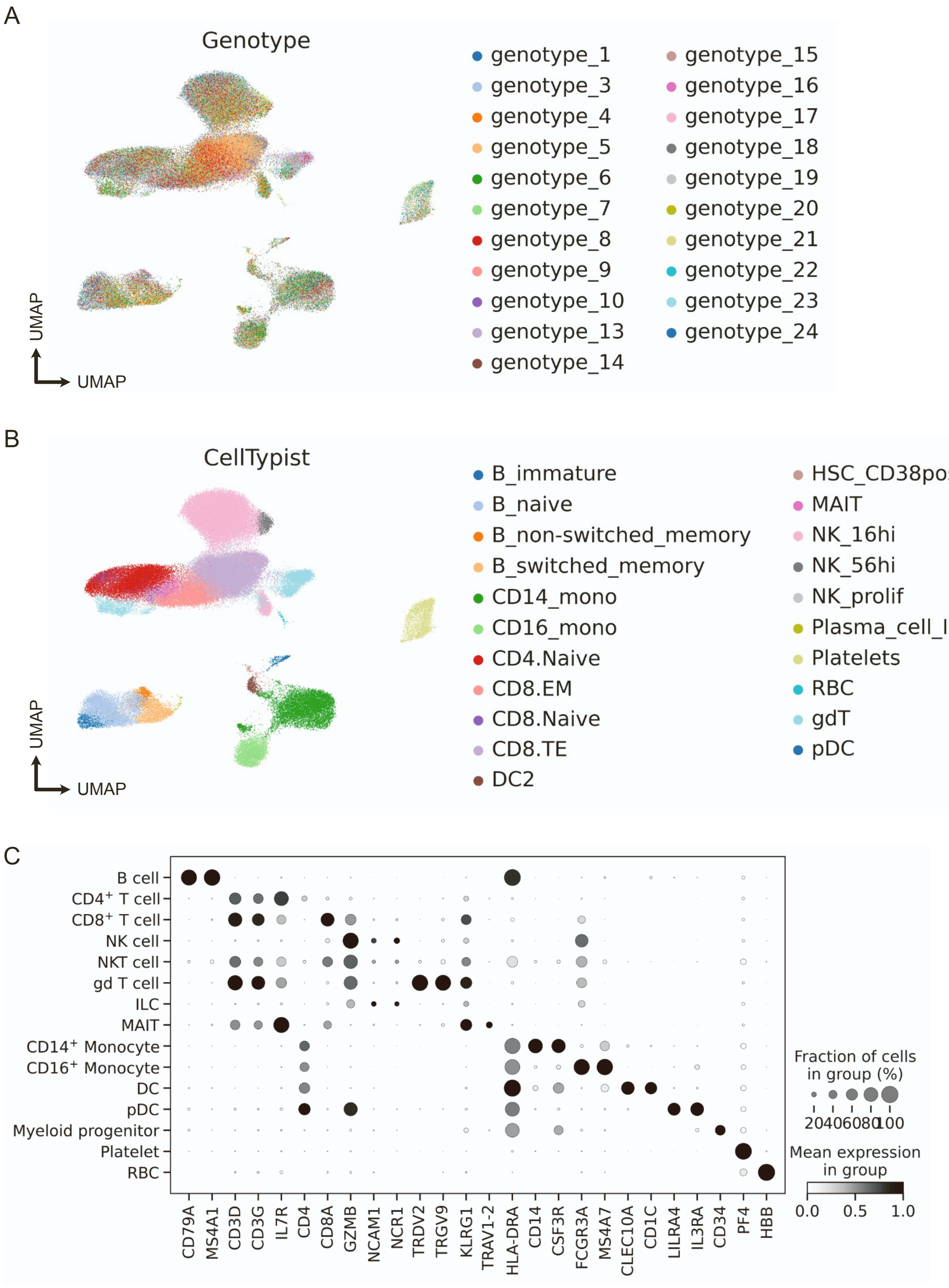

Supplementary figure 7: Supporting data for full scRNAseq dataset. (A) UMAP of all cells captured by scRNAseq post quality control and filtering, by individual genotype. (B) CellTypist annotation of scRNAseq, used for preliminary coarse cell-type identification. (C) Canonical marker gene expression from (A).

Supplementary figure 8

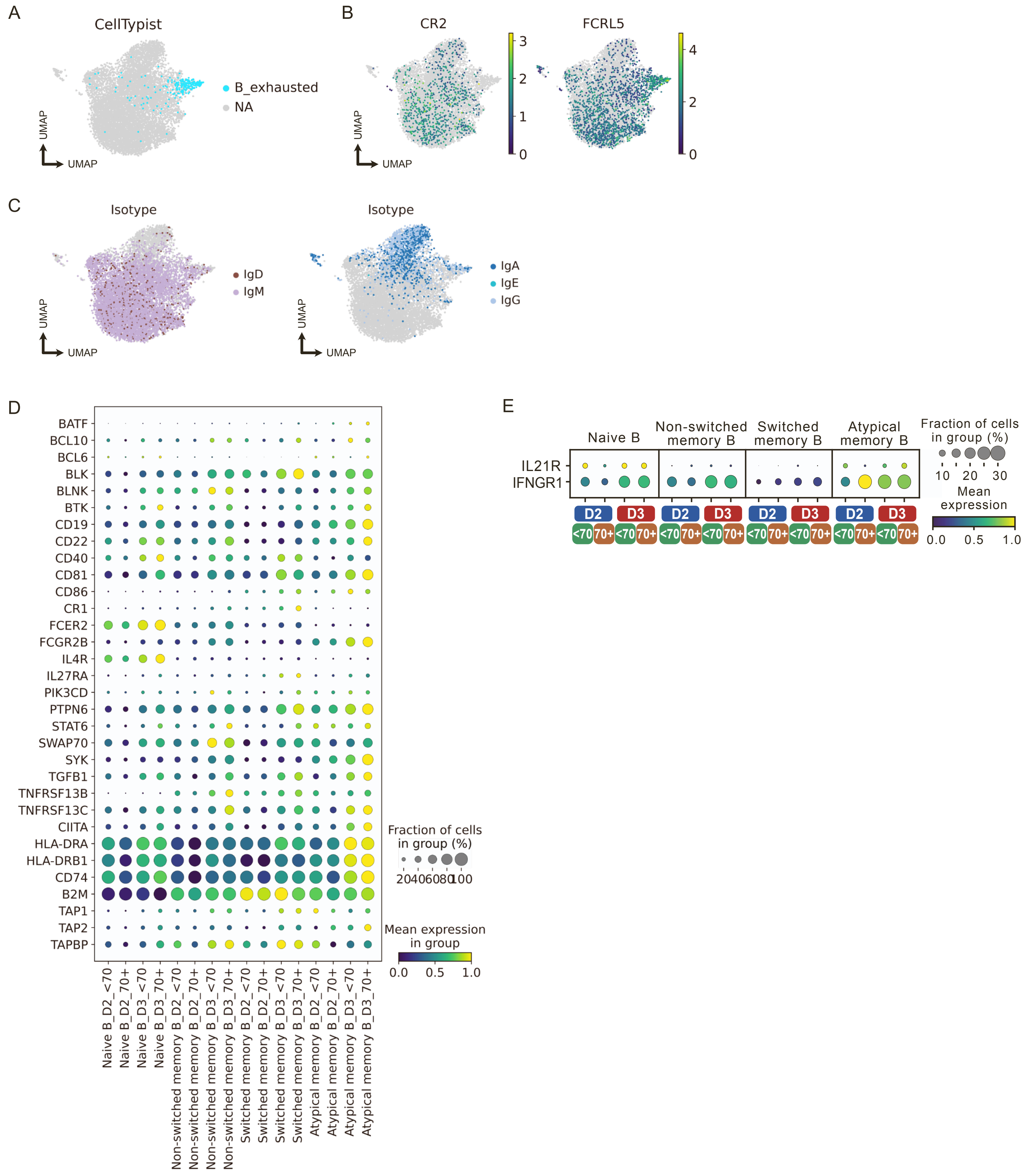

Supplementary figure 8: Supporting data for scRNAseq of B cell subset. (A) CellTypist annotation of atypical memory B cells. (B) Atypical memory B cells expressFCRL5 and lack CR2. (C) Ig heavy-chain isotype calls from matched scBCRseq, used to inform B cell scRNA-seq annotations. (D) Selected differentially expressed genes driving differences in 'Antigen processing and presentation' in B cells, from figure 2G. (E) IL21R and IFNGR1 expression in B cell subsets in <70 and ≥70 individuals 1 month post dose 2 AZD1222 (D2) and 1 month post-mRNA booster (D3).

Supplementary figure 9

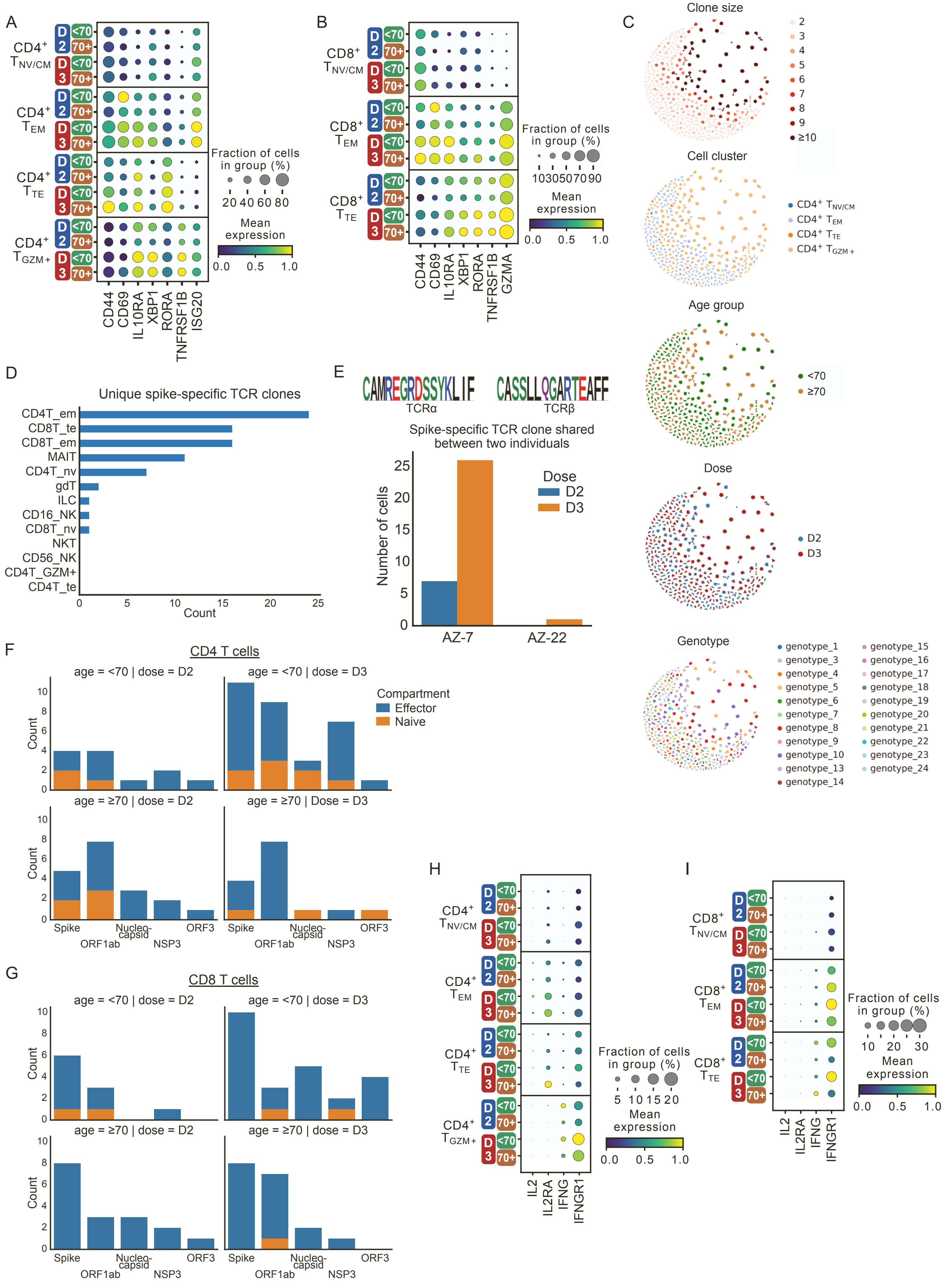

Supplementary figure 9: Supporting data for scTCR/RNAseq of T/NK/ILC cell subset. (A) Selected differentially expressed genes in $CD4^+$ T cell subsets, and $CD8^+$ T cell subsets (B), between <70 and ≥70 individuals or post-D2 and D3. (C) T cell receptor (TCR) network of expanded T cell clones isolated from PBMCs of vaccinated individuals, by clone size, cell subset, age group, timepoint, and genotype (corresponding to individual study participants). (D) Sum of unique spike epitope-specific scTCR clones by corresponding scRNA-seq cell type assignments from Figure 4, across all individuals. (E) Representative example of a SARS-CoV-2 spike epitope-specific TCR clone shared between 2 individuals, AZ-7 (<70) and AZ-22 (≥70). Plot shows TCRα and TCRβ CDR3 sequences (perfect match to TCRβ from IEDB), and number of cells belonging to this clone post-D2 and post-D3. Shared TCR clones between unrelated individuals provide strong support that the TCR clone is vaccine-induced. (F-G) Number of SARS-CoV-2 antigen-specific TCR clones by predicted antigen specificity, in $CD4^+$ and $CD8^+$ T cell subsets respectively. Non-spike specific TCRs were identified in several individuals. (H-I) *IL2*, *IL2RA*, *IFNG*, *IFNGR1* expression in $CD4^+$ and $CD8^+$ T cell subsets respectively, in <70 and ≥70 individuals 1 month post dose 2 AZD1222 (D2) and 1 month post-mRNA booster (D3).

A

B

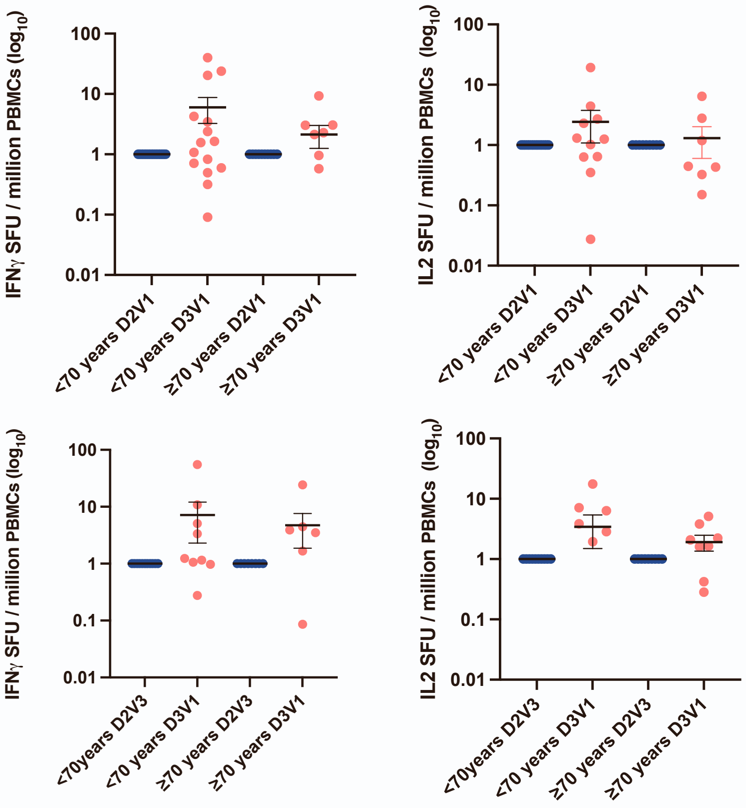

Supplementary Figure 10: Fold changes of IFNy and IL2 release following spike peptide stimulation of PBMC comparing dose 3 with dose 2 in those receiving primary course with two doses of AZD1222 followed by mRNA boost.
(A) Fold changes stratified by age of 1 month post second dose compared to one month post booster for IFNy and IL-2 expression. (B) Fold changes stratified by age of 6 months post second dose and one month post booster for IFNy and IL-2 expression.

Supplementary Table 1: Characteristics of participants receiving two doses of AZD1222 followed by BNT162b2 in the UK who were also N antibody negative at all time points.

	**<70**	**≥70**	**P value**
**n**	23	13	
**Female %**	69.6	69.2	
**Median age (IQR)**	66	73	
**Comorbidities (%)**			
Type 2 Diabetes	2 (8.6)	3 (23)	0.23
History of cancer / immune suppression	5 (22)	1 (7.7)	0.27
Chronic kidney disease	0	1 (7.7)	0.18
Heart disease / hypertension/ high cholesterol	6 (26)	9 (69)	<0.001
**Sera GMT WT**			
D2V1	408.4 (157 - 1063)	266 (111.4 - 635.5)	
D2V3	179.9 (86.11 - 376)	97.68 (35.54 - 268.5)	
D3V1	5749 (1683 - 19633)	1093 (228.8 - 5218)	
**Time between dose 2 & dose 3**	200.5	201.5	ns

Supplementary Table 2: Characteristics of participants receiving three doses of BNT162b2 from Singapore

Gender	Age	Ethnicity
Female	65	Chinese
Male	65	Chinese
Male	65	Chinese
Male	65	Chinese
Male	66	Chinese
Male	66	Chinese
Male	66	Chinese
Male	67	Chinese
Female	67	Other
Male	68	Chinese
Male	68	Chinese
Male	68	Chinese
Female	69	Chinese
Male	69	Chinese
Male	69	Chinese
Male	69	Malay
Female	66	Chinese
Female	68	Malay
Female	70	Chinese
Female	71	Chinese
Female	71	Chinese
Male	71	Chinese
Female	72	Chinese
Female	72	Chinese
Female	72	Chinese
Female	72	Indian
Female	73	Chinese
Male	73	Chinese
Male	73	Chinese
Male	73	Chinese
Male	73	Chinese
Male	73	Chinese
Male	73	Indian
Male	74	Chinese
Male	75	Chinese
Male	76	Chinese

Male	76	Indian
Male	81	Chinese

Supplementary Table 3: Study participants and cell numbers for scRNA-seq

**Age**	**No of Cells post-QC**
71	4414
70	7274
71	6557
75	6825
52	7505
67	5918
54	6479
68	1874
73	6179
74	8206
69	1259
69	6572
67	512
75	4080
74	3301
69	3155
61	2163
62	5577
74	4011
70	3101
62	4422
